# Prevalence of physical frailty, including risk factors, up to 1 year after hospitalisation for COVID-19 in the UK: a multicentre, longitudinal cohort study

**DOI:** 10.1016/j.eclinm.2023.101896

**Published:** 2023-03-11

**Authors:** Hamish J.C. McAuley, Rachael A. Evans, Charlotte E. Bolton, Christopher E. Brightling, James D. Chalmers, Annemarie B. Docherty, Omer Elneima, Paul L. Greenhaff, Ayushman Gupta, Victoria C. Harris, Ewen M. Harrison, Ling-Pei Ho, Alex Horsley, Linzy Houchen-Wolloff, Caroline J. Jolley, Olivia C. Leavy, Nazir I. Lone, William D-C Man, Michael Marks, Dhruv Parekh, Krisnah Poinasamy, Jennifer K. Quint, Betty Raman, Matthew Richardson, Ruth M. Saunders, Marco Sereno, Aarti Shikotra, Amisha Singapuri, Sally J. Singh, Michael Steiner, Ai Lyn Tan, Louise V. Wain, Carly Welch, Julie Whitney, Miles D. Witham, Janet Lord, Neil J. Greening, PHOSP-COVID Study Collaborative Groupai, K. Abel, H. Adamali, D. Adeloye, O. Adeyemi, R. Adrego, L.A. Aguilar Jimenez, S. Ahmad, N. Ahmad Haider, R. Ahmed, N. Ahwireng, M. Ainsworth, B. Al-Sheklly, A. Alamoudi, M. Ali, M. Aljaroof, A.M. All, L. Allan, R.J. Allen, L. Allerton, L. Allsop, P. Almeida, D. Altmann, M. Alvarez Corral, S. Amoils, D. Anderson, C. Antoniades, G. Arbane, A. Arias, C. Armour, L. Armstrong, N. Armstrong, D. Arnold, H. Arnold, A. Ashish, A. Ashworth, M. Ashworth, S. Aslani, H. Assefa-Kebede, C. Atkin, P. Atkin, R. Aul, H. Aung, L. Austin, C. Avram, A. Ayoub, M. Babores, R. Baggott, J. Bagshaw, D. Baguley, L. Bailey, J.K. Baillie, S. Bain, M. Bakali, M. Bakau, E. Baldry, D. Baldwin, M. Baldwin, C. Ballard, A. Banerjee, B. Bang, R.E. Barker, L. Barman, S. Barratt, F. Barrett, D. Basire, N. Basu, M. Bates, A. Bates, R. Batterham, H. Baxendale, H. Bayes, M. Beadsworth, P. Beckett, M. Beggs, M. Begum, P. Beirne, D. Bell, R. Bell, K. Bennett, E. Beranova, A. Bermperi, A. Berridge, C. Berry, S. Betts, E. Bevan, K. Bhui, M. Bingham, K. Birchall, L. Bishop, K. Bisnauthsing, J. Blaikely, A. Bloss, A. Bolger, C.E. Bolton, J. Bonnington, A. Botkai, C. Bourne, M. Bourne, K. Bramham, L. Brear, G. Breen, J. Breeze, A. Briggs, E. Bright, C.E. Brightling, S. Brill, K. Brindle, L. Broad, A. Broadley, C. Brookes, M. Broome, A. Brown, A. Brown, J. Brown, J. Brown, J.S. Brown, M. Brown, M. Brown, V. Brown, T. Brugha, N. Brunskill, M. Buch, P. Buckley, A. Bularga, E. Bullmore, L. Burden, T. Burdett, D. Burn, G. Burns, A. Burns, J. Busby, R. Butcher, A. Butt, S. Byrne, P. Cairns, P.C. Calder, E. Calvelo, H. Carborn, B. Card, C. Carr, L. Carr, G. Carson, P. Carter, A. Casey, M. Cassar, J. Cavanagh, M. Chablani, T. Chalder, J.D. Chalmers, R.C. Chambers, F. Chan, K.M. Channon, K. Chapman, A. Charalambou, N. Chaudhuri, A. Checkley, J. Chen, Y. Cheng, L. Chetham, C. Childs, E.R. Chilvers, H. Chinoy, A. Chiribiri, K. Chong-James, G. Choudhury, N. Choudhury, P. Chowienczyk, C. Christie, M. Chrystal, D. Clark, C. Clark, J. Clarke, S. Clohisey, G. Coakley, Z. Coburn, S. Coetzee, J. Cole, C. Coleman, F. Conneh, D. Connell, B. Connolly, L. Connor, A. Cook, B. Cooper, J. Cooper, S. Cooper, D. Copeland, T. Cosier, M. Coulding, C. Coupland, E. Cox, T. Craig, P. Crisp, D. Cristiano, M.G. Crooks, A. Cross, I. Cruz, P. Cullinan, D. Cuthbertson, L. Daines, M. Dalton, P. Daly, A. Daniels, P. Dark, J. Dasgin, A. David, C. David, E. Davies, F. Davies, G. Davies, G.A. Davies, K. Davies, M.J. Davies, J. Dawson, E. Daynes, A. De Soyza, B. Deakin, A. Deans, C. Deas, J. Deery, S. Defres, A. Dell, K. Dempsey, E. Denneny, J. Dennis, A. Dewar, R. Dharmagunawardena, N. Diar-Bakerly, C. Dickens, A. Dipper, S. Diver, S.N. Diwanji, M. Dixon, R. Djukanovic, H. Dobson, S.L. Dobson, A.B. Docherty, A. Donaldson, T. Dong, N. Dormand, A. Dougherty, R. Dowling, S. Drain, K. Draxlbauer, K. Drury, H.J.C. Drury, P. Dulawan, A. Dunleavy, S. Dunn, C. Dupont, J. Earley, N. Easom, C. Echevarria, S. Edwards, C. Edwardson, H. El-Taweel, A. Elliott, K. Elliott, Y. Ellis, A. Elmer, O. Elneima, D. Evans, H. Evans, J. Evans, R. Evans, R.A. Evans, R.I. Evans, T. Evans, C. Evenden, L. Evison, L. Fabbri, S. Fairbairn, A. Fairman, K. Fallon, D. Faluyi, C. Favager, T. Fayzan, J. Featherstone, T. Felton, J. Finch, S. Finney, J. Finnigan, L. Finnigan, H. Fisher, S. Fletcher, R. Flockton, M. Flynn, H. Foot, D. Foote, A. Ford, D. Forton, E. Fraile, C. Francis, R. Francis, S. Francis, A. Frankel, E. Fraser, R. Free, N. French, X. Fu, J. Fuld, J. Furniss, L. Garner, N. Gautam, J.R. Geddes, J. George, P. George, M. Gibbons, M. Gill, L. Gilmour, F. Gleeson, J. Glossop, S. Glover, N. Goodman, C. Goodwin, B. Gooptu, H. Gordon, T. Gorsuch, M. Greatorex, P.L. Greenhaff, W. Greenhalf, A. Greenhalgh, N.J. Greening, J. Greenwood, H. Gregory, R. Gregory, D. Grieve, D. Griffin, L. Griffiths, A.-M. Guerdette, B. Guillen Guio, M. Gummadi, A. Gupta, S. Gurram, E. Guthrie, Z. Guy, H.H. Henson, K. Hadley, A. Haggar, K. Hainey, B. Hairsine, P. Haldar, I. Hall, L. Hall, M. Halling-Brown, R. Hamil, A. Hancock, K. Hancock, N.A. Hanley, S. Haq, H.E. Hardwick, E. Hardy, T. Hardy, B. Hargadon, K. Harrington, E. Harris, V.C. Harris, E.M. Harrison, P. Harrison, N. Hart, A. Harvey, M. Harvey, M. Harvie, L. Haslam, M. Havinden-Williams, J. Hawkes, N. Hawkings, J. Haworth, A. Hayday, M. Haynes, J. Hazeldine, T. Hazelton, L.G. Heaney, C. Heeley, J.L. Heeney, M. Heightman, S. Heller, M. Henderson, L. Hesselden, M. Hewitt, V. Highett, T. Hillman, T. Hiwot, L.P. Ho, A. Hoare, M. Hoare, J. Hockridge, P. Hogarth, A. Holbourn, S. Holden, L. Holdsworth, D. Holgate, M. Holland, L. Holloway, K. Holmes, M. Holmes, B. Holroyd-Hind, L. Holt, A. Hormis, A. Horsley, A. Hosseini, M. Hotopf, L. Houchen-Wolloff, K. Howard, L.S. Howard, A. Howell, E. Hufton, A.D. Hughes, J. Hughes, R. Hughes, A. Humphries, N. Huneke, E. Hurditch, J. Hurst, M. Husain, T. Hussell, J. Hutchinson, W. Ibrahim, F. Ilyas, J. Ingham, L. Ingram, D. Ionita, K. Isaacs, K. Ismail, T. Jackson, J. Jacob, W.Y. James, W. Jang, C. Jarman, I. Jarrold, H. Jarvis, R. Jastrub, B. Jayaraman, R.G. Jenkins, P. Jezzard, K. Jiwa, C. Johnson, S. Johnson, D. Johnston, C.J. Jolley, D. Jones, G. Jones, H. Jones, H. Jones, I. Jones, L. Jones, M.G. Jones, S. Jones, S. Jose, T. Kabir, G. Kaltsakas, V. Kamwa, N. Kanellakis, S. Kaprowska, Z. Kausar, N. Keenan, S. Kelly, G. Kemp, S. Kerr, H. Kerslake, A.L. Key, F. Khan, K. Khunti, S. Kilroy, B. King, C. King, L. Kingham, J. Kirk, P. Kitterick, P. Klenerman, L. Knibbs, S. Knight, A. Knighton, O. Kon, S. Kon, S.S. Kon, S. Koprowska, A. Korszun, I. Koychev, C. Kurasz, P. Kurupati, C. Laing, H. Lamlum, G. Landers, C. Langenberg, D. Lasserson, L. Lavelle-Langham, A. Lawrie, C. Lawson, C. Lawson, A. Layton, A. Lea, O.C. Leavy, D. Lee, J.-H. Lee, E. Lee, K. Leitch, R. Lenagh, D. Lewis, J. Lewis, K.E. Lewis, V. Lewis, N. Lewis-Burke, X. Li, T. Light, L. Lightstone, W. Lilaonitkul, L. Lim, S. Linford, A. Lingford-Hughes, M. Lipman, K. Liyanage, A. Lloyd, S. Logan, D. Lomas, N.I. Lone, R. Loosley, J.M. Lord, H. Lota, W. Lovegrove, A. Lucey, E. Lukaschuk, A. Lye, C. Lynch, S. MacDonald, G. MacGowan, I. Macharia, J. Mackie, L. Macliver, S. Madathil, G. Madzamba, N. Magee, M.M. Magtoto, N. Mairs, N. Majeed, E. Major, F. Malein, M. Malim, G. Mallison, W. D-C Man, S. Mandal, K. Mangion, C. Manisty, R. Manley, K. March, S. Marciniak, P. Marino, M. Mariveles, M. Marks, E. Marouzet, S. Marsh, B. Marshall, M. Marshall, J. Martin, A. Martineau, L.M. Martinez, N. Maskell, D. Matila, W. Matimba-Mupaya, L. Matthews, A. Mbuyisa, S. McAdoo, H. McAllister-Williams, A. McArdle, P. McArdle, D. McAulay, G.P. McCann, J. McCormick, W. McCormick, P. McCourt, L. McGarvey, C. McGee, K. Mcgee, J. McGinness, K. McGlynn, A. McGovern, H. McGuinness, I.B. McInnes, J. McIntosh, E. McIvor, K. McIvor, L. McLeavey, A. McMahon, M.J. McMahon, L. McMorrow, T. Mcnally, M. McNarry, J. McNeill, A. McQueen, H. McShane, C. Mears, C. Megson, S. Megson, P. Mehta, J. Meiring, L. Melling, M. Mencias, D. Menzies, M. Merida Morillas, A. Michael, C. Miller, L. Milligan, C. Mills, G. Mills, N.L. Mills, L. Milner, S. Misra, J. Mitchell, A. Mohamed, N. Mohamed, S. Mohammed, P.L. Molyneaux, W. Monteiro, S. Moriera, A. Morley, L. Morrison, R. Morriss, A. Morrow, A.J. Moss, P. Moss, K. Motohashi, N. Msimanga, E. Mukaetova-Ladinska, U. Munawar, J. Murira, U. Nanda, H. Nassa, M. Nasseri, A. Neal, R. Needham, P. Neill, S. Neubauer, D.E. Newby, H. Newell, T. Newman, J. Newman, A. Newton-Cox, T. Nicholson, D. Nicoll, A. Nikolaidis, C.M. Nolan, M.J. Noonan, C. Norman, P. Novotny, J. Nunag, L. Nwafor, U. Nwanguma, J. Nyaboko, C. O'Brien, K. O'Donnell, D. O'Regan, L. O’Brien, N. Odell, G. Ogg, O. Olaosebikan, C. Oliver, Z. Omar, P.J.M. Openshaw, L. Orriss-Dib, L. Osborne, R. Osbourne, M. Ostermann, C. Overton, J. Owen, J. Oxton, J. Pack, E. Pacpaco, S. Paddick, S. Painter, A. Pakzad, S. Palmer, P. Papineni, K. Paques, K. Paradowski, M. Pareek, D. Parekh, H. Parfrey, C. Pariante, S. Parker, M. Parkes, J. Parmar, S. Patale, B. Patel, M. Patel, S. Patel, D. Pattenadk, M. Pavlides, S. Payne, L. Pearce, J.E. Pearl, D. Peckham, J. Pendlebury, Y. Peng, C. Pennington, I. Peralta, E. Perkins, Z. Peterkin, T. Peto, N. Petousi, J. Petrie, P. Pfeffer, J. Phipps, J. Pimm, K. Piper Hanley, R. Pius, H. Plant, S. Plein, T. Plekhanova, M. Plowright, K. Poinasamy, O. Polgar, L. Poll, J.C. Porter, J. Porter, S. Portukhay, N. Powell, A. Prabhu, J. Pratt, A. Price, C. Price, C. Price, D. Price, L. Price, L. Price, A. Prickett, J. Propescu, S. Prosper, S. Pugmire, S. Quaid, J. Quigley, J. Quint, H. Qureshi, I.N. Qureshi, K. Radhakrishnan, N.M. Rahman, M. Ralser, B. Raman, A. Ramos, H. Ramos, J. Rangeley, B. Rangelov, L. Ratcliffe, P. Ravencroft, A. Reddington, R. Reddy, A. Reddy, H. Redfearn, D. Redwood, A. Reed, M. Rees, T. Rees, K. Regan, W. Reynolds, C. Ribeiro, A. Richards, E. Richardson, M. Richardson, P. Rivera-Ortega, K. Roberts, E. Robertson, E. Robinson, L. Robinson, L. Roche, C. Roddis, J. Rodger, A. Ross, G. Ross, J. Rossdale, A. Rostron, A. Rowe, A. Rowland, J. Rowland, M.J. Rowland, S.L. Rowland-Jones, K. Roy, M. Roy, I. Rudan, R. Russell, E. Russell, G. Saalmink, R. Sabit, E.K. Sage, T. Samakomva, N. Samani, C. Sampson, K. Samuel, R. Samuel, A. Sanderson, E. Sapey, D. Saralaya, J. Sargant, C. Sarginson, T. Sass, N. Sattar, K. Saunders, R.M. Saunders, P. Saunders, L.C. Saunders, H. Savill, W. Saxon, A. Sayer, J. Schronce, W. Schwaeble, J.T. Scott, K. Scott, N. Selby, M.G. Semple, M. Sereno, T.A. Sewell, A. Shah, K. Shah, P. Shah, M. Shankar-Hari, M. Sharma, C. Sharpe, M. Sharpe, S. Shashaa, A. Shaw, K. Shaw, V. Shaw, A. Sheikh, S. Shelton, L. Shenton, K. Shevket, A. Shikotra, J. Short, S. Siddique, S. Siddiqui, J. Sidebottom, L. Sigfrid, G. Simons, J. Simpson, N. Simpson, A. Singapuri, C. Singh, S. Singh, S.J. Singh, D. Sissons, J. Skeemer, K. Slack, A. Smith, D. Smith, S. Smith, J. Smith, L. Smith, M. Soares, T.S. Solano, R. Solly, A.R. Solstice, T. Soulsby, D. Southern, D. Sowter, M. Spears, L.G. Spencer, F. Speranza, L. Stadon, S. Stanel, N. Steele, M. Steiner, D. Stensel, G. Stephens, L. Stephenson, M. Stern, I. Stewart, R. Stimpson, S. Stockdale, J. Stockley, W. Stoker, R. Stone, W. Storrar, A. Storrie, K. Storton, E. Stringer, S. Strong-Sheldrake, N. Stroud, C. Subbe, C.L. Sudlow, Z. Suleiman, C. Summers, C. Summersgill, D. Sutherland, D.L. Sykes, R. Sykes, N. Talbot, A.L. Tan, L. Tarusan, V. Tavoukjian, A. Taylor, C. Taylor, J. Taylor, A. Te, H. Tedd, C.J. Tee, J. Teixeira, H. Tench, S. Terry, S. Thackray-Nocera, F. Thaivalappil, B. Thamu, D. Thickett, C. Thomas, D.C. Thomas, S. Thomas, A.K. Thomas, T. Thomas-Woods, T. Thompson, A.A.R. Thompson, T. Thornton, M. Thorpe, R.S. Thwaites, J. Tilley, N. Tinker, G.F. Tiongson, M. Tobin, J. Tomlinson, C. Tong, M. Toshner, R. Touyz, K.A. Tripp, E. Tunnicliffe, A. Turnbull, E. Turner, S. Turner, V. Turner, K. Turner, S. Turney, L. Turtle, H. Turton, J. Ugoji, R. Ugwuoke, R. Upthegrove, J. Valabhji, M. Ventura, J. Vere, C. Vickers, B. Vinson, E. Wade, P. Wade, L.V. Wain, T. Wainwright, L.O. Wajero, S. Walder, S. Walker, S. Walker, E. Wall, T. Wallis, S. Walmsley, J.A. Walsh, S. Walsh, L. Warburton, T.J.C. Ward, K. Warwick, H. Wassall, S. Waterson, E. Watson, L. Watson, J. Watson, J. Weir McCall, C. Welch, H. Welch, B. Welsh, S. Wessely, S. West, H. Weston, H. Wheeler, S. White, V. Whitehead, J. Whitney, S. Whittaker, B. Whittam, V. Whitworth, A. Wight, J. Wild, M. Wilkins, D. Wilkinson, B. Williams, N. Williams, N. Williams, J. Williams, S.A. Williams-Howard, M. Willicombe, G. Willis, J. Willoughby, A. Wilson, D. Wilson, I. Wilson, N. Window, M. Witham, R. Wolf-Roberts, C. Wood, F. Woodhead, J. Woods, D.G. Wootton, J. Wormleighton, J. Worsley, D. Wraith, C. Wrey Brown, C. Wright, L. Wright, S. Wright, J. Wyles, I. Wynter, M. Xu, N. Yasmin, S. Yasmin, T. Yates, K.P. Yip, B. Young, S. Young, A. Young, A.J. Yousuf, A. Zawia, L. Zeidan, B. Zhao, B. Zheng, O. Zongo

**Affiliations:** a The Institute for Lung Health, NIHR Leicester Biomedical Research Centre, University of Leicester, Leicester, UK; b University Hospitals of Leicester NHS Trust, Leicester, UK; c University of Nottingham, Nottingham, UK; d Nottingham University Hospitals NHS Trust, Nottingham, UK; e NIHR Nottingham Biomedical Research Centre, Nottingham, UK; f University of Dundee, Ninewells Hospital and Medical School, Dundee, UK; g Centre for Medical Informatics, The Usher Institute, University of Edinburgh, Edinburgh, UK; h MRC Human Immunology Unit, University of Oxford, Oxford, UK; i Oxford University Hospitals NHS Foundation Trust, Oxford, UK; j Division of Infection, Immunity & Respiratory Medicine, Faculty of Biology, Medicine and Health, University of Manchester, Manchester, UK; k Manchester University NHS Foundation Trust, Manchester, UK; l Centre for Exercise and Rehabilitation Science, NIHR Leicester Biomedical Research Centre, University of Leicester, Leicester, UK; m Department of Respiratory Sciences, University of Leicester, Leicester, UK; n Therapy Department, University Hospitals of Leicester, NHS Trust, Leicester, UK; o Centre for Human & Applied Physiological Sciences, School of Basic & Medical Biosciences, Faculty of Life Sciences & Medicine, King's College London, London, UK; p Department of Respiratory Medicine, King's College Hospital NHS Foundation Trust, London, UK; q Department of Health Sciences, University of Leicester, Leicester, UK; r Royal Infirmary of Edinburgh, NHS Lothian, Edinburgh, UK; s Royal Brompton and Harefield Clinical Group, Guy's and St Thomas' NHS Foundation Trust, UK; t National Heart and Lung Institute, Imperial College London, London, UK; u Faculty of Life Sciences and Medicine, King's College London, UK; v Department of Clinical Research, London School of Hygiene & Tropical Medicine, London, UK; w Hospital for Tropical Diseases, University College London Hospital, London, UK; x Division of Infection and Immunity, University College London, London, UK; y University of Birmingham, Birmingham, UK; z University Hospital Birmingham NHS Foundation Trust, Birmingham, UK; aa Asthma and Lung UK, London, UK; ab Division of Cardiovascular Medicine, Radcliffe Department of Medicine, University of Oxford, Oxford, UK; ac NIHR Leicester Biomedical Research Centre, University of Leicester, Leicester, UK; ad Leeds Institute of Rheumatic and Musculoskeletal Medicine, University of Leeds, Leeds, UK; ae NIHR Leeds Biomedical Research Centre, Chapel Allerton Hospital, Leeds, UK; af The School of Life Course & Population Sciences, King's College London, UK; ag AGE Research Group, NIHR Newcastle Biomedical Research Centre, Newcastle University, Newcastle upon Tyne, UK; ah Newcastle upon Tyne Hospitals NHS Foundation Trust, Newcastle upon Tyne, UK

**Keywords:** COVID-19, Physical frailty, Long-COVID, Fried's frailty phenotype, Hospitalisation

## Abstract

**Background:**

The scale of COVID-19 and its well documented long-term sequelae support a need to understand long-term outcomes including frailty.

**Methods:**

This prospective cohort study recruited adults who had survived hospitalisation with clinically diagnosed COVID-19 across 35 sites in the UK (PHOSP-COVID). The burden of frailty was objectively measured using Fried's Frailty Phenotype (FFP). The primary outcome was the prevalence of each FFP group—robust (no FFP criteria), pre-frail (one or two FFP criteria) and frail (three or more FFP criteria)—at 5 months and 1 year after discharge from hospital. For inclusion in the primary analysis, participants required complete outcome data for three of the five FFP criteria. Longitudinal changes across frailty domains are reported at 5 months and 1 year post-hospitalisation, along with risk factors for frailty status. Patient-perceived recovery and health-related quality of life (HRQoL) were retrospectively rated for pre-COVID-19 and prospectively rated at the 5 month and 1 year visits. This study is registered with ISRCTN, number ISRCTN10980107.

**Findings:**

Between March 5, 2020, and March 31, 2021, 2419 participants were enrolled with FFP data. Mean age was 57.9 (SD 12.6) years, 933 (38.6%) were female, and 429 (17.7%) had received invasive mechanical ventilation. 1785 had measures at both timepoints, of which 240 (13.4%), 1138 (63.8%) and 407 (22.8%) were frail, pre-frail and robust, respectively, at 5 months compared with 123 (6.9%), 1046 (58.6%) and 616 (34.5%) at 1 year. Factors associated with pre-frailty or frailty were invasive mechanical ventilation, older age, female sex, and greater social deprivation. Frail participants had a larger reduction in HRQoL compared with before their COVID-19 illness and were less likely to describe themselves as recovered.

**Interpretation:**

Physical frailty and pre-frailty are common following hospitalisation with COVID-19. Improvement in frailty was seen between 5 and 12 months although two-thirds of the population remained pre-frail or frail. This suggests comprehensive assessment and interventions targeting pre-frailty and frailty beyond the initial illness are required.

**Funding:**

10.13039/100014013UK Research and Innovation and 10.13039/501100000272National Institute for Health Research.

**Research in context**

**Evidence before this study**

We searched PubMed for studies investigating the burden of frailty in hospitalised survivors of COVID-19 using the search terms (“frailty” AND “covid-19” AND “hospital∗”). Several studies examined and confirmed the relationship between frailty prior to COVID-19 illness and adverse outcomes during hospitalisation while others examined the effect of critical care admission with COVID-19 and the transition to a frail state. No cohort studies of hospitalised survivors examining the burden of frailty in this group were identified, furthermore no investigation of the association of objectively assessed physical frailty and ongoing symptoms and physical performance was identified.

**Added value of this study**

To our knowledge we report the only cohort of hospitalised survivors of COVID-19 in whom physical frailty was objectively assessed as a pre-defined outcome measure. The cohort highlights an important and considerable burden of frailty and pre-frailty, which is predictive of adverse outcomes in the medium-to-long term. Our data suggest that both pre-existing frailty and acute worsening from the hospitalisation contribute to the high proportion of pre-frail and frail people at 5 months. The data support the physical frailty model in identifying and guiding the ongoing management of COVID-19 survivors in whom further interventions may be beneficial, and is associated with non-recovery, ongoing symptoms, reduced physical performance and reduced health-related quality of life.

**Implications of all the available evidence**

Frailty is a common finding in survivors of COVID-19 requiring hospitalisation, with our data extending the findings to beyond the critical care setting. We suggest routine assessment of frailty in the post-hospitalised population, such as the comprehensive geriatric assessment, and the development of targeted interventions.

## Introduction

As of October 2022, more than 1 million individuals^1^ have been admitted to hospitals in the UK with COVID-19. Survival from hospitalisation has improved throughout the pandemic with increasingly successful interventions.^2^ The longer-term consequences of severe COVID-19, including sequelae of the primary infection and the effects of prolonged hospitalisation and immobility and reduced habitual physical activity in daily living, are now emerging. Perceived muscle weakness and fatigue are reported as the most common symptoms, present in 63% of patients at six months in those hospitalised with COVID-19.^3^ Despite the primary pathology affecting the lungs, it is clear that there are multiple systemic consequences of COVID-19 and therefore a more global assessment is required.^4^

Frailty describes a clinical syndrome characterised by increased vulnerability to stressors due to a loss of in-built body reserves.^5^ Multiple operationalised models of frailty have been utilised including that described by Fried et al.,^6^ who defined the phenotype model as the presence of core identifiable clinical features of physical frailty which correlate with an increased risk of adverse outcomes including death, hospitalisation, falls and developing a new disability and is recommended as an objective measure of physical frailty.^7^

Hospitalisation with severe COVID-19 is more common in patients with frailty, and is a known risk factor for inpatient mortality, more so than the presence of co-morbidities or increasing age.^8^ Among patients who survive hospitalisation, many will have had prolonged immobility, and may have received either invasive ventilatory support in an intensive care (ICU) setting, or non-invasive ventilatory support in a non-ICU setting. In other hospitalised populations, frailty is a surrogate marker to identify individuals who are at an increased risk of medium-to-long term adverse outcomes including hospital readmission, loss of independence and need for further care and support. There is an urgent need to understand the longer-term burden of frailty among individuals recovering from COVID-19, along with potential mechanisms to identify and guide interventions for patients with, or at risk.

The “Post-hospitalisation COVID-19 study” (PHOSP-COVID) is a UK-wide national consortium deep phenotyping cohort study, designed to understand long-term health outcomes in this population. In our cohort we aimed to: (1) describe the burden of physical pre-frailty and frailty using Fried's frailty phenotype model (FFP) and the change in frailty status between 5 months and 1 year; (2) identify risk factors associated with the presence of pre-frailty and frailty; (3) identify potential targets for interventions using the sub-domains of the FFP; (4) understand changes in health-related quality of life and self-reported recovery across frailty groups.

## Methods

### Study design and population

PHOSP-COVID is a prospective longitudinal cohort study recruiting adult participants who have been admitted to an admissions unit or ward at a UK hospital and discharged with a diagnosis of confirmed or suspected COVID-19 between 5th March 2020 and 31st March 2021.^9^ Recruitment occurred across 83 hospital sites in England, Scotland, Wales and Northern Ireland with eligible participants being invited to participate by each site following discharge. Patients were excluded if they had a confirmed diagnosis of a pathogen unrelated to the objectives of the study, attended an accident and emergency department but were not admitted, or had another life-limiting illness with life expectancy of less than 6 months. Study methods have previously been described.^9^ Data from participants’ hospital admission and up to two research visits the first occurring between two and seven months (“5-month visit”) and second more than 10 months (“1-year visit”) following discharge were included. Participants in whom there was a minimum dataset for evaluation of FFP criteria at their 5-month visit were included.

### Ethics

All participants provided written informed consent. The study was approved by the Leeds West Research Ethics Committee (20/YH/0225) and registered with ISRCTN, number ISRCTN10980107.

### Outcome measures

Outcome measures were assessed at each participating site. Patient centred tests were performed by delegated site study staff who had received video-based training alongside study specific standard operating procedures for each assessment.

The primary outcome was the prevalence of each FFP group; robust (no FFP criteria), pre-frail (one or two FFP criteria) and frail (three or more FFP criteria) at 5 months and 1 year following discharge from hospital. The assessment and cut off values used to assess each of the five FFP criteria (unintentional weight loss, weakness, exhaustion, slowness and low physical activity) are described in detail in Supplementary Table ST1. For inclusion in the primary analysis participants required complete outcome data for three of the five FFP criteria as previously described by Fried.^6^

Key measures included demographics at time of hospitalisation for COVID-19, including age, ethnicity, working status prior to hospitalisation, length of hospital stay, co-morbidities and severity of acute illness (WHO Clinical Progression Scale)^10^ and quintiles for index of multiple deprivation.^11^ Patient-reported outcome measures at the research visit included patient-perceived recovery and health-related quality of life (HRQoL) both prospectively at the study visit and retrospectively rated for before onset of COVID-19 illness (EQ5D-5L Utility Index).^12^

### Statistical analysis

Baseline characteristics were analysed using either one-way analysis of variance (ANOVA) or Kruskal–Wallis H-test to compare parametric and non-parametric continuous data, respectively, and categorical data using the chi-squared test. Data distribution was assessed visually using histograms for each variable. Missing data were reported for each outcome measure with no imputation for modelling. The presence of no frailty, pre-frailty and frailty at 5 months and 1 year post discharge was modelled using mixed methods ordinal logistic regression with pre-defined clinically plausible independent variables assessed initially in univariable ordinal logistic regression models for significant interaction. Clinically similar variables were assessed for collinearity using visual inspection of plotted values and the Pearson correlation coefficient for continuous data and plotting of means of continuous data grouped by ordinal categories and Kendall's tau-b test, both with a threshold of r > 0.5 implying collinearity. Variables were selected for inclusion in a final multivariable model using a significance threshold of p < 0.05 providing they were not collinear. Variables assessed were: age at admission (as a continuous variable and in decade blocks); sex; body mass index (BMI) (as a continuous variable and as four ordinal categories); severity of COVID-19 (in four ordinal categories defined using the WHO Clinical Progression Scale)^13^; duration of hospitalisation for COVID-19 (as a continuous variable); number of co-morbidities prior to hospitalization (as a three level ordinal variable); social deprivation (using quintiles of the index of multiple deprivation); and time to assessment from discharge (as a continuous variable). Testing for heteroscedasticity was performed by plotting predicted values against residuals.

Among participants with data at both time points who changed FFP category a sub-analysis was performed across four discrete groups: frail at 5 months to pre-frail/robust at 1 year, pre-frail at 5 months to robust at 1 year, pre-frail at 5 months to frail at 1 year and robust to pre-frail/frail at 1 year. Change in HRQoL using EQ5D-5L utility index^12^ over the three time points (prior to COVID, 5 months and 1 year) and between frailty groups was assessed using a repeated measures mixed model.

Two sensitivity analyses were performed; the first among those in whom all five FFP sub-domains were available to assess potential bias from missing data within the cohort, the second in participants aged over 65 years to assess the representativeness of the FFP model in our younger population.

No sample size calculation was performed for the assessment of physical frailty as an outcome measure in the study. Significance was determined using p values of less than 0.05 without adjustment for multiple testing. Analysis was conducted using STATA 16.0 (StataCorp, TX) within the Public Health Scotland National Safe Haven platform.

### Role of the funding source

The funders of the study had no role in study design, data collection, data analysis, data interpretation, or writing of the report.

## Results

### Participant characteristics

**Fig. 1:**
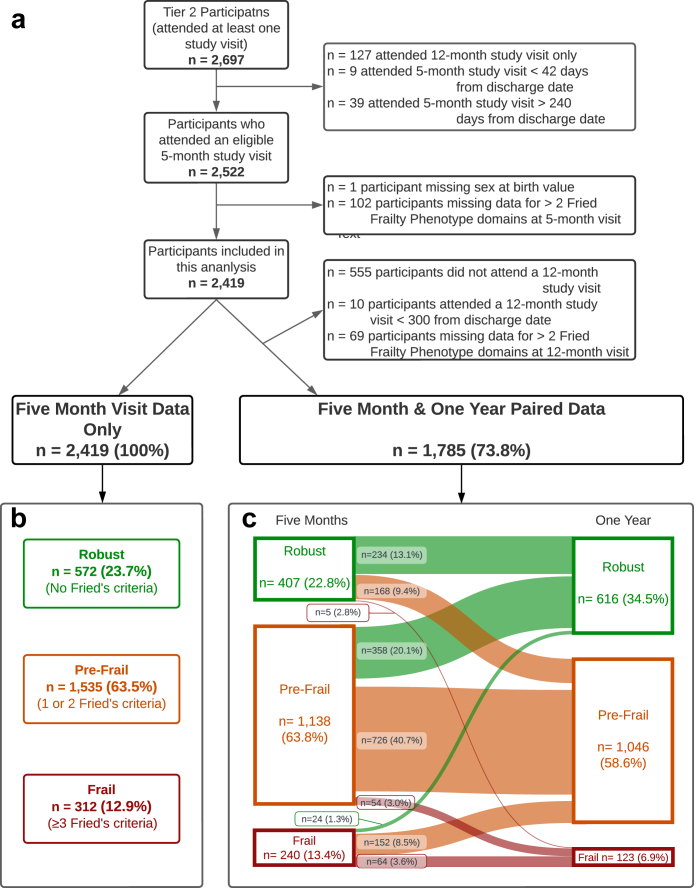
**Study profile.** (a) The number of participants included and reasons for exclusion. (b) Frailty domain proportions at 5 months. (c) Frailty domain proportions at 5 months and 1 year, with movements between frailty domains shown.

Between March 5, 2020, and March 31, 2021, 2697 participants consented to attend study visits of whom 2419 (89.7%) with sufficient data for FFP assessment at 5 months from 35 study sites were included in this analysis (Fig. 1). Participants’ 5-month assessment visit occurred at mean 5.3 (SD 1.5) months following hospital discharge. A 1-year assessment occurred for 1785/2419 (73.8%), with visits occurring at mean 12.7 (SD 1.16) months following hospital discharge. The mean age of all included participants was 57.9 (SD 12.7) years, 933/2419 (38.6%) were female and 1795/2419 (74.6%) reported being of white ethnicity (Table 1). Participants represented a spectrum of acute illness severity categories with 429/2419 (17.7%) requiring invasive mechanical ventilation and 392/2419 (16.2%) requiring admission to hospital without supplemental oxygen. Differences in baseline characteristics were noted between those participants who attended both visits compared with those who missed the 1 year assessment, notably, participants who missed the 1 year visit were younger (mean age 55.1 [SD 13.7] vs 58.8 [12.11] years [p < 0.001]), had undergone a shorter initial hospital stay (median 7 [IQR 4–13] vs 8 [4–16] days [p = 0.009]) and a lower proportion were of white ethnicity (419/634 [66.9%] vs 1376/1785 [77.3%] [p < 0.001]) compared to those attending both visits. Other baseline characteristics were similar between these two groups (Table 1). It was not possible to confirm the survival status at 1 year of all participants who did not attend their 1 year visit at the time of analysis due to delays in healthcare data linkage however analysis of the subset of participants in the PHOSP-COVID study who were also recruited the ISARIC study (roughly 60% of the cohort) suggested that the 5 month to 1 year mortality rate was around 1% as seen in other survivor cohorts.^14^

**Table 1:** Baseline and hospital admission characteristics.

Frailty status at 1 year	Participants with both 5 month and 1 year data (n = 1785 [73.8%])									Participants with 5 month data only (n = 634 [26.2%])	All study participants (n = 2419 [100%])	p value
	Robust		Pre-frail		Frail		All participants		p value			
**N (%)**	616	(34.5%)	1046	(58.6%)	123	(6.89%)	1785	(100%)				
**Age at admission (years)**^a,^^c^	55.5	12.2	60.2	11.6	63.9	12.3	58.8	12.1	<0.001	55.1 (13.7)	57.9 (12.6)	<0.001
**Body mass index (kg/m**^**2**^**)**^a,^^c^	32.5	8.07	32.6	7.59	33.6	8.12	32.6	7.79	0.355	32.9 (8.57)	32.7 (7.99)	0.423
**Length of hospital stay (days)**^b^	7	4–14	8	4–17	10	6–24	8	4–16	<0.001	7 (4–13)	8 (4–15)	0.009
**Sex**									0.008			0.667
Female	215	(34.9%)	418	(40.0%)	60	(48.8%)	693	(38.8%)		240 (37.9%)	933 (38.6%)	
Male	401	(65.1%)	628	(60.0%)	63	(51.2%)	1092	(61.2%)		394 (62.1%)	1486 (61.4%)	
**Ethnicity**^c^									0.126			<0.001
White	483	(78.9%)	806	(77.1%)	87	(70.7%)	1376	(77.3%)		419 (66.9%)	1795 (74.6%)	
South Asian	67	(10.9%)	97	(9.28%)	15	(12.2%)	179	(10.1%)		104 (16.6%)	283 (11.8%)	
Black	33	(5.39%)	77	(7.37%)	9	(7.32%)	119	(6.69%)		54 (8.63%)	173 (7.19%)	
Mixed	11	(1.80%)	24	(2.30%)	2	(1.63%)	37	(2.08%)		15 (2.40%)	52 (2.16%)	
Other	18	(2.94%)	41	(3.92%)	10	(8.13%)	69	(3.88%)		34 (5.43%)	103 (4.28%)	
**Number of co-morbidities**									<0.001			0.069
None	195	(31.7%)	211	(20.2%)	14	(11.4%)	420	(23.5%)		176 (27.8%)	596 (24.6%)	
1 comorbidity	154	(25.0%)	215	(20.6%)	17	(13.8%)	386	(21.6%)		140 (22.1%)	526 (21.7%)	
2+ comorbidities	267	(43.3%)	620	(59.3%)	92	(74.8%)	979	(54.8%)		318 (50.2%)	1297 (53.6%)	
**Working status before COVID-19 illness**									<0.001			0.633
Working full or part time^c^	362	(70.2%)	421	(47.8%)	36	(32.7%)	819	(54.3%)		274 (55.6%)	1093 (54.7%)	
**WHO clinical progression scale**									<0.001			0.079
WHO class 4 (no oxygen therapy)	93	(15.1%)	159	(15.2%)	17	(13.8%)	269	(15.1%)		123 (19.4%)	392 (16.2%)	
WHO class 5 (oxygen by mask or nasal prongs)	282	(45.8%)	427	(40.8%)	58	(47.2%)	767	(43.0%)		266 (42.0%)	1033 (42.7%)	
WHO class 6 (oxygen by non-invasive ventilation or high flow nasal oxygen)	152	(24.7%)	259	(24.8%)	14	(11.4%)	425	(23.8%)		140 (22.1%)	565 (23.4%)	
WHO class 7–9 (admitted to ICU for intubation and mechanical ventilation)	89	(14.4%)	201	(19.2%)	34	(27.6%)	324	(18.2%)		105 (16.6%)	429 (17.7%)	

All n(%) except ^a^Mean [SD] and ^b^Median [IQR]. Percentages are calculated by category after exclusion of missing data for that variable.

c Missing data (age n < 5, body mass index n = 167, ethnicity n = 13, working before COVID-19 illness n = 419).

A sub-analysis comparing baseline characteristics of the 1643 participants with complete data for all five FFP sub-domains compared to the 776 with data for only three or four sub-domains is shown in the Supplementary Appendix; Supplementary Fig. SF5a–c. Similar proportions were seen for all characteristics except the number of participants in full or part time work prior to their COVID-19 illness for which a higher proportion (56.4% vs 50.8%, p = 0.0210) seen among those with no missing FFP sub-domain data.

### Frequency and characteristics of Fried's frailty phenotype

At 5 months following discharge, 572/2419 (23.7%) of participants were robust, 1535/2419 (63.5%) were pre-frail (one or two criteria), and 312/2419 (12.9%) of participants were frail (three or more criteria) (Fig. 1b). In participants with paired data (both 5 months and 1 year) proportions in each FFP group were similar to those with data only at 5 months (Fig. 1b and c). At 1 year there was a significant improvement in frailty status, with the proportion who were robust increasing to 616/1785 (34.5%) while the pre-frail and frail groups reducing to 1046/1785 (58.6%) and 123/1785 (6.89%), respectively (p < 0.001). Only a small proportion 54/1138 (4.75%) of those who were pre-frail at 5 months progressed to being frail at 1 year compared with 176/240 (73.3%) of those who were frail at 5 months whose phenotype improved to pre-frail or robust (Fig. 1c).

The demographics of participants in each FFP group at 1 year are displayed in Table 1 highlighting that participants living with frailty were significantly older (mean age 63.9 [SD 12.3] years vs 60.2 [11.6] and 55.5 [12.2] years [p < 0.001]) and more likely to have two or more comorbidities (92/123 [74.8%] vs 620/1046 [58.6%] and 267/616 [43.3%] [p < 0.001]) than those in the pre-frail and robust groups, respectively, at 1 year with similar differences seen at 5 months except for sex which showed that the proportion of frail and pre-frailty among men was lower than women at 1 year (p = 0.008) but this was not seen at 5 months (Supplementary Table ST2).

Frail participants were less likely to have been in work before their COVID-19 illness (p < 0.001). Participants in the frail and pre-frail groups had a longer hospital stay than those who were not frail (p < 0.001). The proportion of participants intubated and ventilated was highest in the frail group (p < 0.001).

### Risk factors associated with frailty post-hospitalisation

Individual, clinically plausible risk factors for frailty were identified at 5 months and 1 year (univariable regression results Supplementary Table ST3). Duration of hospitalisation had significant co-linearity with WHO Clinical Progression Scale (Kendall's tau-b = 0.57, p < 0.001) and was excluded from the multivariable model. There was no evidence of heteroscedasticity and residuals were normally distributed.

**Fig. 2:**
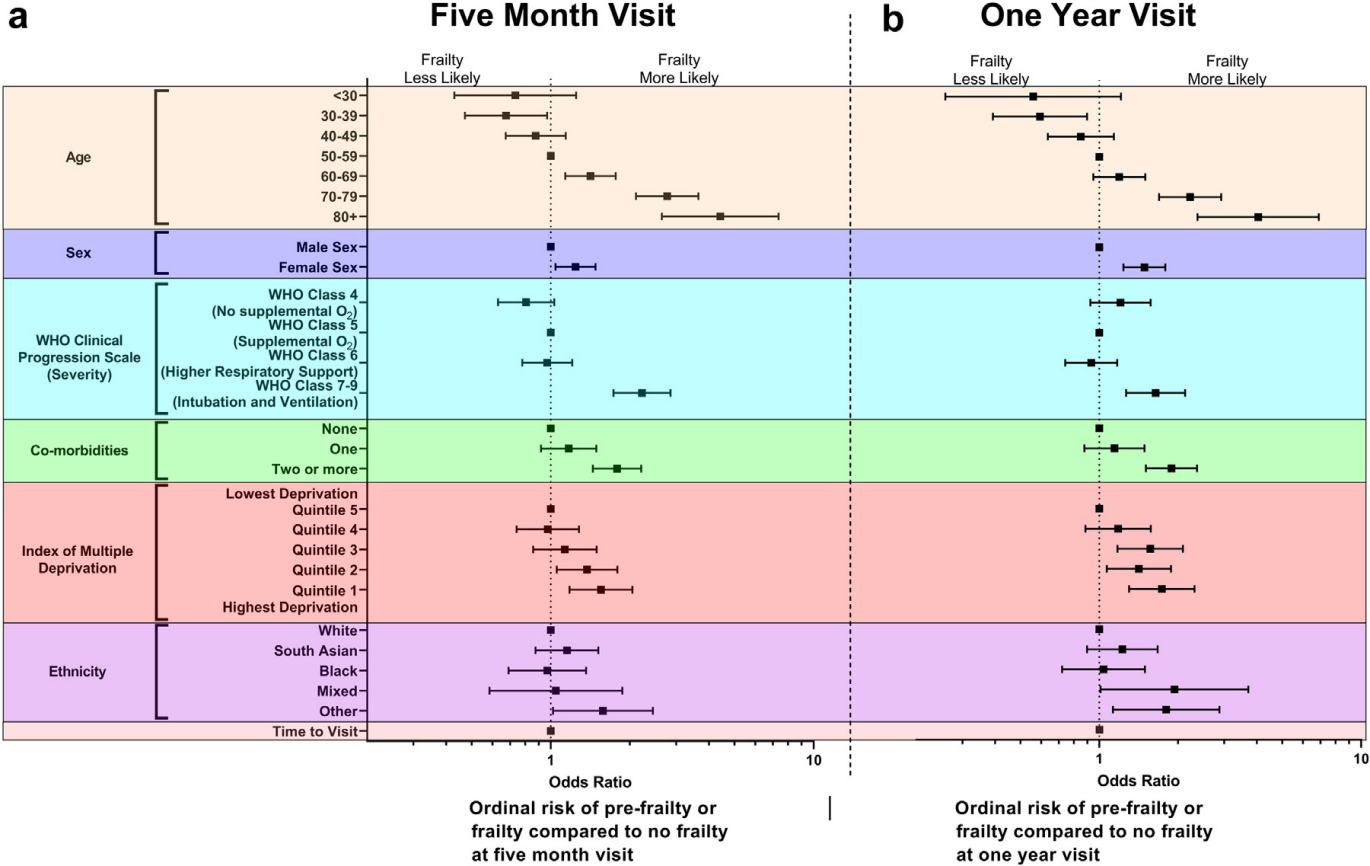
**Risk factors for the presence of frailty at (a) 5 months and (b) 1 year following hospitalisation for COVID-19.** Data presented are odds ratios and 95% confidence intervals using multivariable ordinal regression. Numerical values are shown in Supplementary Table ST5.

Older age, the presence of two or greater comorbidities, the requirement for intubation and ventilation at the time of COVID illness, female sex and higher social deprivation were all significant risk factors for the presence of frailty at both 5 months and 1 year following hospitalisation (Fig. 2 and Supplementary Table ST4). Time since discharge was included as a co-variate in the model and hospital site as a random effect. The risk factors were similar at both time points.

Sensitivity analyses for these risk factors were completed both for missing FFP sub-domains (Supplementary Appendix; Supplementary Fig. SF5b) and among participant over the age of 65 years only (Supplementary Appendix; Supplementary Fig. SF6b) with similar risk factor patterns seen in both sub-groups.

### Change in frailty status between 5 months and 1 year

Overall 534/1785 (29.9%) of individuals improved their frailty status between 5 months and 1 year while 227/1785 (12.7%) had a decline in their frailty status leaving 1024/1785 (57.4%) unchanged (p < 0.001) (Fig. 1c). Whilst numerically the largest change was an improvement between being pre-frail at 5 months to being robust at 1 year, the highest proportional change from a group at 5 months was among the frail group, where 176/240 (73.3%) of individuals were re-assessed as either robust or pre-frail at 1 year.

**Fig. 3:**
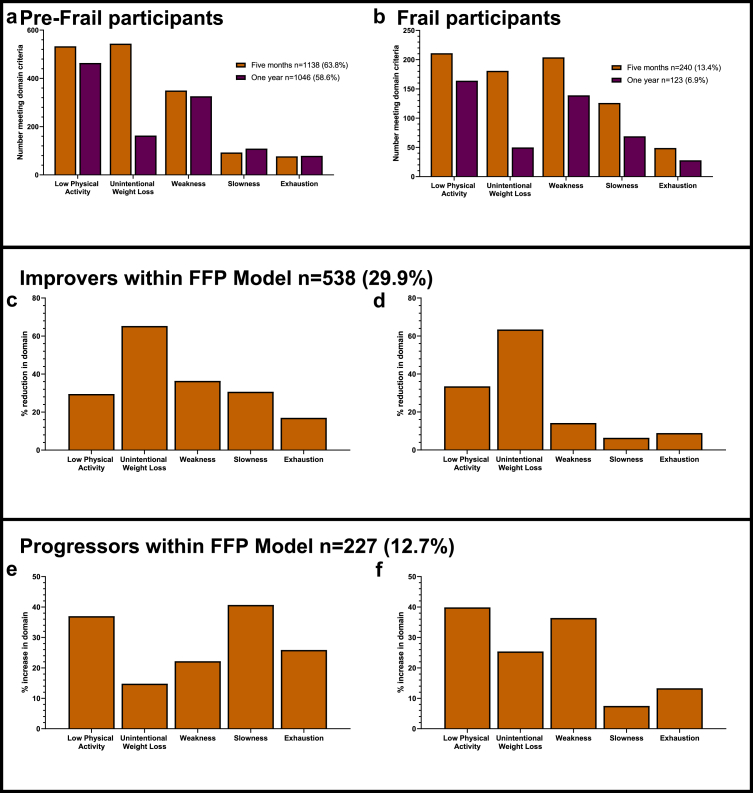
**Frailty status.** (a) Number of participants meeting criteria of each FFP sub-domain in those classified as pre-frail at 5 months and 1 year. (b) Number of participants meeting criteria of each FFP sub-domain in those classified as frail at 5 months and 1 year. (c) Percent fall in participants meeting criteria for each FFP sub-domain among those who improved from frail to either pre-frail or robust between 5 months and 1 year (n = 176). (d) Percent fall in participants meeting criteria for each FFP sub-domain among those who improved from pre-frail to robust between 5 months and 1 year (n = 358). (e) Percent increase in participants meeting criteria for each FFP sub-domain among those who progressed from pre-frail to frail between 5 months and 1 year (n = 54). (f) Percent increase in participants meeting criteria for each FFP sub-domain among those who progressed from robust to either pre-frail or frail between 5 months and 1 year (n = 173). FFP=Fried's Frailty Phenotype.

To investigate the drivers of change in frailty status over time, changes within the sub-domains were explored: in both the pre-frail and frail groups the proportion of people reporting unintentional weight loss fell significantly between 5 months and 1 year (544/1138 [47.8%] vs 163/1046 [15.6%] for pre-frail and 181/240 [75.4%] vs 50/123 [40.7%] for frail groups, both p < 0.001) (Fig. 3a and b).

The proportion of individuals meeting each FFP domain are shown for those who improved (Fig. 3c and d) and those who progressed (Fig. 3e and f). While changes in improvers were most commonly due to a reduction in unintentional weight loss, reduced rates of weakness and slow walking speed were also seen, particularly among those improving from the frail group at 5 months.

Participants improving from being pre-frail at 5 months to robust at 1 year were younger (mean (SD) age 56.9 [SD 12.0] vs 60.4 [12.0] years [p < 0.001]), more likely to have no co-morbidities (32.1% vs 18.7% [p < 0.001]) and also more likely to be male (67.9% vs 59.4% [p = 0.006]) than those who remained pre-frail or progressed to being frail.

Among individuals whose frailty status progressed from pre-frail to frail (Fig. 3e) the most common two domains that increased were low physical activity and slowness, whereas for those who progressed from robust to pre-frail (Fig. 3f) it was in the low physical activity and weakness domains. Participants who progressed from being pre-frail to frail were older (mean age 62.7 [SD 12.7] vs 59.1 [11.9] years [p = 0.030]), a higher proportion were female (53.7% vs 37.2% [p = 0.015]) and a lower proportion had no co-morbidities (9.3% vs 23.6% [p = 0.002]) than those who remained pre-frail or who became robust by 1 year. Similarly, those who progressed from robust at 5 months to pre-frail or frail at 1 year were older (mean age 57.0 [SD 11.3] vs 53.3 [11.8] years [p = 0.001]) and had a lower BMI (mean BMI 31.56 [6.27] vs 33.49 [9.45] kg/m^2^ [p = 0.023]) than those who remained robust.

Further exploration of the factors that may have driven changes in FFP status between 5 months and 1 year may be revealed within our sensitivity analysis by acute illness (Supplementary Appendix; Supplementary Fig. SF7a–c). Here, a similar proportion of participants were classified as pre-frail at 5 months (56.9%, 65.4%, 65.9% and 62.7%) and 1 year (59.1%, 55.7%, 60.9% and 62.0%) across the four groups (WHO class 4, no supplemental oxygen; WHO class 5, oxygen by mask or nasal prongs; WHO class 6, oxygen by NIV or HFNO; WHO class 7–9, admitted to ICU for intubation and mechanical ventilation). WHO class 6 has the lowest proportion of participants being frail at 1 year (3.3%) (Supplementary Fig. SF7c) while the smallest change in frailty from 5 months to 1 year is seen in the least severe WHO class 4 groups (Supplementary Appendix; Supplementary Fig. SF7a). The largest reduction in the proportion of frail participants was seen in the most severe WHO class 7–9 group where this proportion fell from 23.5% at 5 months to 10.5% at 1 year (Supplementary Appendix; Supplementary Fig. SF7d).

Further sensitivity analyses for missing data (Supplementary Appendix; Supplementary Fig. SF5a–c) and among participants aged over 65 years (Supplementary Appendix; Supplementary Fig. SF6a and b) were performed with similar patterns of frailty seen in these sub-groups to the full cohort.

### Health-related quality of life and self-reported recovery from COVID-19

**Fig. 4:**
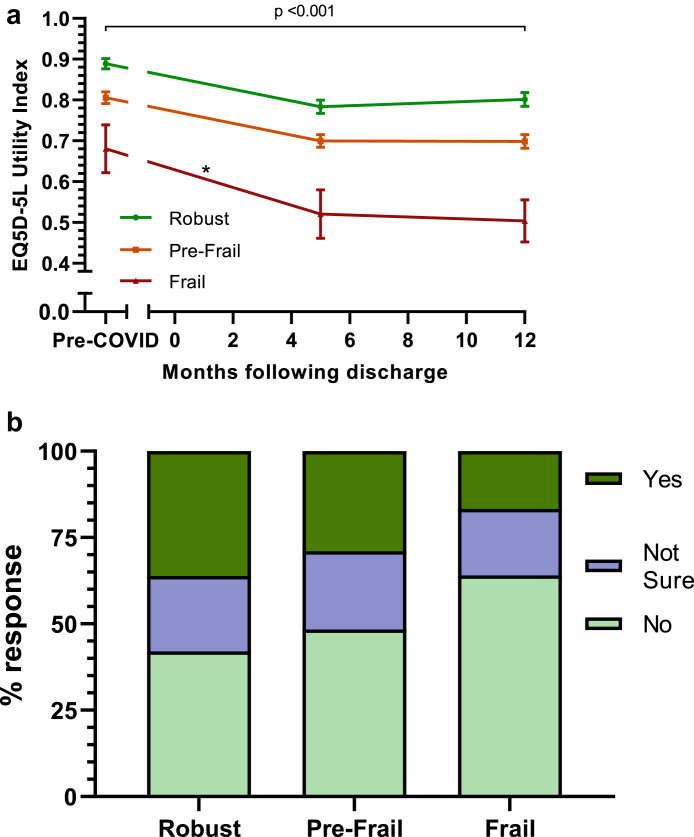
**HQQoL and recovery.** (a) Change in HRQoL (assessed via EQ5D-5L Utility Index) from pre-COVID to 5 month and 1 year visits by frailty status at 1 year. ∗Change from baseline to 5 months greater in the frail group than robust group p = 0.004. (b) Self-reported recovery at 1 year visit, by frailty status at 1 year. HRQoL = health-related quality of life.

Individuals who were frail at 1 year had a lower health-related quality of life (HRQoL), assessed using the EQ5D-5L utility index, at each time point including prior to their COVID-19 illness compared with those who were pre-frail or robust (mean 0.680 [95% CI 0.058] vs 0.806 [0.015] and 0.889 [0.013], respectively). All groups reported a significant fall in HRQoL from prior to COVID at 5 months, which remained low at 1 year (Fig. 4a) with those in the frail group falling significantly more than those in the pre-frail and robust groups at both 5 months (p = 0.004).

A minority of participants reported being fully recovered following COVID-19 at 1 year in all FFP groups though this was significantly lower among participants living with frailty at 1 year with 19/114 (16.7%) reporting feeling recovered compared with 265/912 (29.1%) in the pre-frail and 180/498 (36.1%) robust groups at 1 year (p < 0.001) (Fig. 4b).

## Discussion

In this study of hospitalised COVID-19 survivors we show that both physical frailty and pre-frailty are common at 1 year following discharge, together remaining present in two-thirds of participants. While some recovery is seen between 5 months and 1 year we identified that those least likely to recover were older, more likely to be female, have been treated in ICU, have multiple co-morbidities and live in an area with higher levels of deprivation. Those who remain frail or pre-frail at 1 year are more likely to report that they have not recovered from their acute illness, while those who are frail suffered a greater and more persistent fall in their HRQoL from prior to hospitalisation for COVID-19. Our data suggest reduced levels of physical activity are a driver for at least some individuals with new or persistent frailty. Although frailty is a concept usually associated with adverse outcomes in older people, our study population were predominantly working age people with 55% working either full or part time prior to their hospitalisation, emphasising that this high prevalence is likely to have an impact on income and economic activity as well as disability and quality of life.

The burden of pre-frailty and frailty in this cohort is significantly higher than in a similarly-aged population from the UK Biobank, in which 38% were pre-frail and 3% frail.^15^ While hospitalised survivors of COVID-19 are unlikely to be representative of the general population, the true prevalence of frailty pre-COVID-19 in this cohort is unknown. Patients with frailty are more likely to be admitted to hospital, so are likely to comprise a higher proportion of our population. However, survival from severe illness is also lower in patients with frailty,^16^ potentially reducing the frequency of frailty in our cohort, as attendance at 5 months was required. The nature of our study design prevents a causal relationship from being established, however the population represents a large sub-group identified as being at risk for adverse health outcomes and thus who may benefit from further risk stratification.

We were able to identify a number of independent risk factors for the presence of physical frailty. These point to a likely combination of probable pre-existing frailty (suggested by older age, multiple co-morbidities and higher social deprivation), with higher risk of admission, as well as newly-acquired frailty following COVID-19 (as suggested by the requirement for invasive mechanical ventilation). The impact of admission to ICU on longer-term function has been known for a number of years,^17^^,^^18^ with significant muscle loss seen very rapidly from admission.^19^ It is therefore not surprising that mechanical ventilation carried an acute risk for frailty with over a quarter of participants in this group categorised as frail at 1 year following discharge. It is worth noting that this association is less likely to be mediated by pre-existing frailty, given that pre-existing frailty was a criteria used for ICU rationing during COVID-19 in the UK.^20^^,^^21^ Furthermore the finding that those admitted to ICU had both the highest proportion of frailty at 1 year as well as the highest proportion improving from the frail category to the pre-frail or robust categories suggests that this may be predominantly newly acquired frailty with potential for a dynamic response during recovery and further highlighting the role of the acute critical illness in promoting frailty in this cohort as seen in other studies.^22^ This could potentially be even more pronounced in COVID-19 than other critical illnesses given the long hospital stay and prolonged ventilation time seen during the pandemic.^23^ However, 80% of participants in this cohort did not require intubation and those requiring supplemental oxygen represented the largest proportion of frail participants at 1 year.

It is also important to consider the interaction of both chronic state and the acute event. Our data suggest that not only did those with frailty at 1 year have a lower HRQoL before their COVID-19 illness, they also experienced the largest self-reported fall in this measure between pre-COVID and both follow up timepoints. This would fit with increased vulnerability to acute stressors that underpins frailty, putting this group at a high risk of worsening frailty.

Our analysis of the groups whose frailty status changes between 5 months and 1 year suggest a role for reduced physical activity in driving the development or persistence of frailty in this cohort with those progressing from being robust to being pre-frail demonstrating increased levels of low physical activity and weakness whereas increased frequency of slow walking speed is predominantly seen among those progressing from being pre-frail to frail. This may suggest reduced activity as a precursor to slower walking speed and the development of frailty and potentially highlighting a target for interventions to improve outcomes in this cohort. Similarly, the participants whose FFP improved during the follow up period showed a marked reduction in low physical activity. Furthermore, the high proportion of individuals with weakness and low physical activity^15^ suggests that individuals in this cohort may have a particular phenotype of pre-frailty and frailty that has been shown to be associated with a poorer prognosis^24^ and who may benefit more from targeted exercise interventions. Other potential mechanistic factors not explored here but which have been seen in post-COVID populations include prolonged inflammation^25^ and both persistent cardiac^26^ and respiratory damage^27^ all of which may contribute to reduced physical performance and the cycle of exhaustion and inactivity.

Among those that improved their frailty status, the largest change in FFP domain was seen in a reduction in unexplained weight loss. While weight may have stabilised, or increased, between 5 months and 1 year it is not known whether this represents change in either muscle mass or fat mass. Given reports of lack of functional improvement at 12 months,^25^ this would potentially suggest predominantly fat gain, which may carry additional future risk.

There are well described benefits of carefully tailored exercise and rehabilitation in addressing functional deficit in patients with chronic disease^28^^,^^29^ and evidence supporting the use of telerehabilitation following COVID-19 is emerging.^30^ Given the pattern of reduced exercise performance and strength in this cohort it is possible that a similar benefit will be seen, though ongoing studies seek to confirm this.^31^ It is noteworthy that concerns about post-exertional malaise in patients who have not fully recovered from COVID-19 have been raised.^32^

Our data highlight a large unmet need for the majority of patients who have been admitted to hospital with COVID-19. The high burden of both pre-frailty and frailty with limited recovery between 5 months and 1 year demonstrates the need for healthcare follow-up with a holistic assessment and potential for treatable traits. While COVID-19 is a novel disease and has resulted in unprecedented strain on healthcare systems, longer-term assessment of its impact could use strategies from other specialities that address multidimensional aspects of health. For example, screening patients with the Clinical Frailty Scale and then performing an adapted Comprehensive Geriatric Assessment may be appropriate^33^ to help identify limitations and guide therapies, though there may be practical limitations to the widespread use of this in a clinical setting where other surrogate measures such as the Timed Up and Go or Sit to Stand tests may provide useful screening alternatives.^34^^,^^35^ These therapies may need to address both risk to future events, as well as patient-orientated goals.^36^

There are limitations to this study. The nature of severe COVID-19 and the study design meant that it was not possible to obtain objective measures of frailty prior to the acute illness. We used the EQ5D-5L as a marker of pre-illness HRQoL, though this was completed retrospectively and may be subject to recollection bias. We screened for the clinical frailty scale in the medical records at the time of admission but this was documented in fewer than 15% of cases. We also used patient-perceived recovery to understand individuals’ perceived baseline. Our use of the FFP model for assessing frailty provides a robust, clinically relevant and comparable measure of physical frailty over and above solely functional measures of activities of daily living. Although the original model was validated in a cohort of older adults aged over 65 years,^6^ multiple more recent studies have validated its use in younger populations and our sub-analysis of participants over 65 years suggests similar risk factors remain applicable in that age group.^37^ Furthermore, the absence of comparison control groups either admitted to hospital for other reasons or who suffered COVID-19 illness without hospitalisation limits the assessment of causality. With regard to the cohort reported in this analysis, conclusions can only be drawn with the context of survivors of an evolving pandemic illness for which acute treatments changed during the course of the study. A “healthy dropout” effect appears to have been present with younger participants with a shorter duration of hospitalization being more likely to miss their 1 year study assessment which also prevented the use of multiple imputation in our modelling as missing data was unlikely to be missing at random.

In summary, we have shown in our cohort that pre-frailty and frailty are present in the majority of people who have survived hospital admission from COVID-19 up to 1 year following discharge with evidence of recovery in some individuals from 5 months to 1 year. Clinical assessment and development of proven interventions targeting the wider effects, including frailty, are needed in this large patient group, both to support those living with frailty and help prevent the development of frailty among the large pre-frail group.

## Contributors

HJCM and NJG conducted the analysis and drafted the initial manuscript. HJCM, RAE, CEB, JDC, OE, L-PH, AH, MM, BR, LVW, SJS and NJG contributed to the conception or design of the work; the acquisition of data for the work; and the analysis, or interpretation of data for the work. LH-W, ASi and AShi contributed to the conception or design of the work; and the acquisition of data for the work. KP and JML contributed to the conception or design of the work; and the analysis, or interpretation of data for the work. CEBo, AG, CJJ, OCL, WD-CM, DP, MSe, MSt and CW contributed to the acquisition of data for the work; and the analysis, or interpretation of data for the work. VCH and RMS contributed to the acquisition of data for the work. ABD, PLG, EMH, NIL, JKQ, MR, ALT, JW and MDW contributed to the analysis, or interpretation of data for the work. All authors contributed to data interpretation and critical review and revision of the manuscript, gave final approval of the version to be published, and agree to be accountable for all aspects of the work in ensuring that questions related to the accuracy or integrity of any part of the work are appropriately investigated and resolved. HJCM and NJG have accessed and verified the data. HJCM and NJG were responsible for the decision to submit the manuscript.

## Data sharing statement

The protocol, consent form, definition and derivation of clinical characteristics and outcomes, training materials, regulatory documents, information about requests for data access, and other relevant study materials are available online at https://www.phosp.org/

## Declaration of interests

RAE declares speaker fees or support from Boeringher Ingelheim and 10.13039/100019719Chiesi as well as their role as ERS Group 01.02 Pulmonary Rehabilitation Secretary. CEBo and AG declare support from 10.13039/501100020624NIHR Nottingham BRC and Nottingham university Hospitals Trust R&I and Nottingham Hospitals Charity. CEB declares grants and consultancy paid to institution from GSK, AZ, 10.13039/100004339Sanofi, 10.13039/100001003Boeringher Ingelheim, Chiesi, 10.13039/100004336Novartis, 10.13039/100004337Roche, 10.13039/100004328Genentech, Mologic, 4DPharma outside of this work. JDC declares grants from 10.13039/100004337Roche, Insmed, 10.13039/100001003Boehringer Ingelheim, 10.13039/100004336Novartis, GSK, 10.13039/100005564Gilead Sciences and 10.13039/501100016387Grifols as well as consulting fees from 10.13039/100004325Astrazeneca, Insmed, 10.13039/100001003Boehringer Ingelheim, Janssen, Chiesi, 10.13039/100004336Novartis, Grifols, Zambon, Pfizer and GSK outside of this work. AH declares institutional and individual support from the NIHR Manchester BRC as well as their role as Deputy Chair NIHR Translational Research Collaboration. WD-CM declares grants or contracts from National Institute for Health Research, Asthma + Lung UK and NHSX as well as payment or honoraria for lectures, presentations, speakers bureaus, manuscript writing or educational events from Mundipharma, 10.13039/100004336Novartis and European Conference and Incentive Services and participation on a Data Safety Monitoring Board or Advisory Board for Jazz Pharmaceuticals and receipt of equipment, materials, drugs, medical writing, gifts or other services from GSK for funds for blood analysis outside of this work. JKQ declares grants paid to institution from the Medical Research Council, HDR UK, GSK, Bayer, 10.13039/100001003Boehringer Ingelheim, Asthma + Lung UK, 10.13039/100019719Chiesi and 10.13039/100004325AstraZeneca as well as advisory bord participation or speaking fees from 10.13039/100004330GlaxoSmithKline, 10.13039/100001003Boehringer Ingelheim, 10.13039/100004325AstraZeneca, 10.13039/100019719Chiesi, Teva, Insmed and 10.13039/100004326Bayer. BR declares consulting fees from Axcella Therapeutics outside of this work. SJS declares grants from the NIHR programme grant (NIHR 202020) as an NIHR Senior Investigator, the Wellcome Doctoral Training Programme, HTA Project Grant (NIHR: 131015), NIHR DHSC/UKRI COVID-19 Rapid Response Initiative, NIHR Global Research Group (NIHR 17/63/20) and Actegy Limited, honoraria paid by GSK, Ministry of Justice, CIPLA and Sherbourne Gibbs for presentations, participation on the NICE Expert Adviser Panel - Long COVID and Wales Long COVID Advisory Board (expired) and a role as ATS Pulmonary Rehabilitation Assembly Chair, Clinical Lead RCP Pulmonary Rehabilitation Accreditation Scheme and Clinical Lead NACAP Audit for Pulmonary Rehabilitation. LVW declares additional support from GSK and 10.13039/501100000362Asthma + Lung UK (Professorship (C17-1)) relating to this work as well as research funding from Orion Pharma and GSK, research collaboration contracts from Genentech and 10.13039/100004325AstraZeneca, consulting fees paid to institution from Galapagos for participation in an advisory board, support for travel from Genentech and their role as Associate Editor for European Respiratory Journal outside of this work. JW declares grants from the 10.13039/501100009130NIHR programme development grant and 10.13039/100015544King’s College Hospital Charity as well as participation on the trial steering group for “Falls in Care Homes (FinCH)” and programme steering group member for “Promoting Activity, Independence and Stability in Early Dementia (PrAISED) research programme” as their role as Clinical lead for national audit of inpatient falls (Royal College of Physicians) outside of this work. NJG declares grants or contracts from the NIHR, GSK and MRC, consulting fees paid to institution from Genentech, lecture fees from 10.13039/100004325AstraZeneca and 10.13039/100001003Boehringer Ingelheim as well as both lecture fees and support for travel from 10.13039/100019719Chiesi outside of this work. HJCM, ABD, OE, PLG, VCH, EMH, L-PH, LH-W, CJJ, OCL, NIL, MM, DP, KP, MR, RMS, MSe, AShi, ASi, MSt, ALT, CW, MDW and JML declare no competing interests.

## Appendix A Supplementary data

Supplementary Tables ST1–ST4 and Figures SF5–SF7

PHOSP_Collaborative_Group_Authors
