# Supplementary material for: Prevalence of physical frailty, including risk factors, up to 1 year after hospitalisation for COVID-19 in the UK: a multicentre, longitudinal cohort study
Source: eClinicalMedicine. 2023 Mar 11;57:101896. doi: 10.1016/j.eclinm.2023.101896 (PMC10005893; doi:10.1016/j.eclinm.2023.101896)
Supplement: Supplementary Tables ST1–ST4 and Figures SF5–SF7 [file mmc1.docx]

**Supplementary Material**

Table of Contents

[Supplementary Table ST1: Criteria used to define Frailty within the cohort 2](#_Toc126594005)

[Supplementary Table ST2: Baseline characteristics across FFP groups at 5 months and 1 year 3](#_Toc126594006)

[Supplementary Table ST3: Univariable ordinal regression for risk of frailty at 5 months and 1 year following hospitalisation 5](#_Toc126594007)

[Supplementary Table ST4: Multivariable ordinal regression for risk of frailty at 5 months and 1 year following hospitalisation 6](#_Toc126594008)

[Supplementary Figure SF5a: Sensitivity analysis for missing data in the FFP model: Number of participants in each FFP group at 5 months and 1 year including numbers moving between groups during this interval among those with data available for all 5 FFP criteria at both time points. 7](#_Toc126594009)

[Supplementary Figure SF5b: Sensitivity analysis for missing data in the FFP model: Multivariable ordinal regression for risk of frailty at 5 months and 1 year following hospitalisation among those with data available for all 5 FFP criteria at both time points. 8](#_Toc126594010)

[Supplementary Table ST5c: Sensitivity analysis for missing data in the FFP model: Comparison of baseline characteristics between those with data available for only three of 5 compared to all 5 FFP criteria at both time points. 9](#_Toc126594011)

[Supplementary Figure SF6a: Sensitivity analysis for participants age ≥65 years only: Number of participants in each FFP group at 5 months and 1 year including numbers moving between groups during this interval among participants age ≥65 years only. 10](#_Toc126594012)

[Supplementary Figure SF6b: Sensitivity analysis for participants age ≥65 years only: Multivariable ordinal regression for risk of frailty at 5 months and 1 year following hospitalisation among participants age ≥65 years only. 11](#_Toc126594013)

[Supplementary Figure SF7a-d: Sensitivity analysis of transition of participants between frailty status by WHO Severity during acute illness. 12](#_Toc126594014)

[References: 13](#_Toc126594015)

## Supplementary Table ST1: Criteria used to define Frailty within the cohort

|  | **Domain** | **Measure** | | | |
| --- | --- | --- | --- | --- | --- |
| **Fried’s**  **Frailty**  **Phenotype (FFP)*** | “Unintentional weight loss” | Assessed subjectively with the question:^1^ | “In the last year, has the patient lost more than 10lb (4·5 kilograms) unintentionally?” | | = "Yes" |
|  | “Weakness” | Assessed objectively by isometric hand grip strength with the strongest of up to three attempts in each hand. Cut off values were stratified by sex and BMI.^1^ † | | Men (BMI <=24) | <=29 Kg |
|  |  |  |  | Men (24<BMI<=28) | <=30 Kg |
|  |  |  |  | Men (BMI >28) | <=32 Kg |
|  |  |  |  | Women (BMI <=23) | <=17 Kg |
|  |  |  |  | Women (23<BMI<=26) | <=17·3 Kg |
|  |  |  |  | Women (26<BMI<=29) | <=18 Kg |
|  |  |  |  | Women(BMI >29) | <=21 Kg |
|  | “Exhaustion” | Assessed subjectively from participant responses to two questions:^2^ | “Do you feel full of energy? | | (Yes/No) = "No" |
|  |  |  | “During the last 4 weeks how often have you rested in bed during the day?” | | (Not at all/Once/Every day/Every Week) = "Every day" or "Every Week" |
|  | Slowness | Assessed objectively using the faster of up to two efforts of four metre gait speed:^2^ † | | Men with height <= 1·73m | >=6·124s (speed <= 0·653 m/s) |
|  |  |  |  | Men with height > 1·73m | >=5·249s (speed <=0·762 m/s) |
|  |  |  |  | Women with height <=1·59m | >=6·124s (speed <= 0·653 m/s) |
|  |  |  |  | Women with height > 1·59m | >=5·249s (speed <=0·762 m/s) |
|  | Low Physical Activity | Assessed objectively using the validated General Practice Physical Activity Questionnaire scoring of General Activity Index.^3^ | | | = “Inactive” |

*Specific cut-off values used for each domain matched those originally used by Fried *et al.*^1^ where possible and are annotated by

## Supplementary Table ST2: Baseline characteristics across FFP groups at 5 months and 1 year

|  | Frailty status at 5 months | | | | | | | | | | Frailty status at 1 year | | | | | | | | |
| --- | --- | --- | --- | --- | --- | --- | --- | --- | --- | --- | --- | --- | --- | --- | --- | --- | --- | --- | --- |
|  | Robust | | Pre-Frail | | | Frail | | All With Frailty Data | | p value | Robust | | Pre-Frail | | Frail | | All With Frailty Data | | p value |
| **Denominator** | **572** | **23·6%** | **1535** | **63·5%** | **312** | | **12·9%** | **2419** | **100·0%** |  | **616** | **34·5%** | **1046** | **58·6%** | **123** | **6·9%** | **1785** | **100·0%** |  |
| **Age at admission (years)*^** | **54·07** | (12·1) | **58·20** | (12·6) | **63·05** | | (11·8) | **57·85** | (12·6) | 0·0000 | **55·49** | (12·2) | **60·18** | (11·6) | **63·94** | (12·3) | **58·82** | (12·1) | 0·0000 |
| **Body Mass Index (kg/m^2^)*^** | **32·61** | (8·17) | **32·54** | (7·61) | **33·62** | | (9·21) | **32·70** | (7·99) | 0·0964 | **32·49** | (8·07) | **32·59** | (7·59) | **33·59** | (8·12) | **32·63** | (7·79) | 0·3552 |
| **Hospital Stay (days)** **†** | **6·00** | (3-11) | **8·00** | (4-15) | **13·00** | | (6-31) | **8·00** | (4-15) | 0·0001 | **7·00** | (4-14) | **8·00** | (4-17) | **10·00** | (6-24) | **8·00** | (4-16) | 0·0001 |
| **Age Category** |  |  |  |  |  | |  |  |  | 0·0000 |  |  |  |  |  |  |  |  | 0·0000 |
| <30 | 23 | **(4·03%)** | 35 | **(2·28%)** | <5 | | **(<2%)** | 61 | **(2·52%)** |  | 14 | **(2·27%)** | 13 | **(1·24%)** | 0 | **(0·00%)** | 27 | **(1·51%)** |  |
| 30-39 | 51 | **(8·93%)** | 96 | **(6·25%)** | <5 | | **(<2%)** | 151 | **(6·24%)** |  | 56 | **(9·09%)** | 46 | **(4·40%)** | <5 | **(<3%)** | 105 | **(5·88%)** |  |
| 40-49 | 110 | **(19·3%)** | 227 | **(14·8%)** | 36 | | **(11·5%)** | 373 | **(15·4%)** |  | 110 | **(17·9%)** | 121 | **(11·6%)** | 14 | **(11·4%)** | 245 | **(13·7%)** |  |
| 50-59 | 192 | **(33·6%)** | 437 | **(28·5%)** | 69 | | **(22·1%)** | 698 | **(28·9%)** |  | 198 | **(32·1%)** | 297 | **(28·4%)** | 27 | **(22·0%)** | 522 | **(29·2%)** |  |
| 60-69 | 148 | **(25·9%)** | 446 | **(29·1%)** | 105 | | **(33·7%)** | 699 | **(28·9%)** |  | 171 | **(27·8%)** | 337 | **(32·2%)** | 34 | **(27·6%)** | 542 | **(30·4%)** |  |
| 70-79 | 40 | **(7·01%)** | 251 | **(16·4%)** | 73 | | **(23·4%)** | 364 | **(15·1%)** |  | 57 | **(9·25%)** | 203 | **(19·4%)** | 32 | **(26·0%)** | 292 | **(16·4%)** |  |
| 80+ | 7 | **(1·23%)** | 43 | **(2·80%)** | 22 | | **(7·05%)** | 72 | **(2·98%)** |  | 10 | **(1·62%)** | 29 | **(2·77%)** | 13 | **(10·6%)** | 52 | **(2·91%)** |  |
| **Sex** |  |  |  |  |  | |  |  |  | 0·2760 |  |  |  |  |  |  |  |  | 0·0080 |
| Female | 222 | **(38·8%)** | 577 | **(37·6%)** | 134 | | **(42·9%)** | 933 | **(38·6%)** |  | 215 | **(34·9%)** | 418 | **(40·0%)** | 60 | **(48·8%)** | 693 | **(38·8%)** |  |
| Male | 350 | **(61·2%)** | 958 | **(62·4%)** | 178 | | **(57·1%)** | 1486 | **(61·4%)** |  | 401 | **(65·1%)** | 628 | **(60·0%)** | 63 | **(51·2%)** | 1092 | **(61·2%)** |  |
| **Ethnicity** |  |  |  |  |  | |  |  |  | 0·0200 |  |  |  |  |  |  |  |  | 0·1260 |
| White | 422 | **(74·0%)** | 1154 | **(75·7%)** | 219 | | **(70·2%)** | 1795 | **(74·6%)** |  | 483 | **(78·9%)** | 806 | **(77·1%)** | 87 | **(70·7%)** | 1376 | **(77·3%)** |  |
| South Asian | 81 | **(14·2%)** | 167 | **(11·0%)** | 35 | | **(11·2%)** | 283 | **(11·8%)** |  | 67 | **(10·9%)** | 97 | **(9·3%)** | 15 | **(12·2%)** | 179 | **(10·1%)** |  |
| Black | 35 | **(6·14%)** | 109 | **(7·15%)** | 29 | | **(9·29%)** | 173 | **(7·19%)** |  | 33 | **(5·39%)** | 77 | **(7·37%)** | 9 | **(7·32%)** | 119 | **(6·69%)** |  |
| Mixed | 12 | **(2·11%)** | 35 | **(2·30%)** | 5 | | **(1·60%)** | 52 | **(2·16%)** |  | 11 | **(1·80%)** | 24 | **(2·30%)** | <5 | **(<2%)** | 37 | **(2·08%)** |  |
| Other | 20 | **(3·51%)** | 59 | **(3·87%)** | 24 | | **(7·69%)** | 103 | **(4·28%)** |  | 18 | **(2·94%)** | 41 | **(3·92%)** | 10 | **(8·13%)** | 69 | **(3·88%)** |  |
| **Co-morbidities (List)** |  |  |  |  |  | |  |  |  |  |  |  |  |  |  |  |  |  |  |
| Cardiovascular | 219 | **(38·3%)** | 717 | **(46·7%)** | 176 | | **(56·4%)** | 1112 | **(46·0%)** | 0·0000 | 239 | **(38·8%)** | 530 | **(50·7%)** | 72 | **(58·5%)** | 841 | **(47·1%)** | 0·0000 |
| Nero/Physch | 88 | **(15·4%)** | 319 | **(20·8%)** | 82 | | **(26·3%)** | 489 | **(20·2%)** | 0·0060 | 93 | **(15·1%)** | 222 | **(21·2%)** | 43 | **(35·0%)** | 358 | **(20·1%)** | 0·0060 |
| Respiratory | 132 | **(23·1%)** | 412 | **(26·8%)** | 109 | | **(34·9%)** | 653 | **(27·0%)** | 0·0010 | 146 | **(23·7%)** | 290 | **(27·7%)** | 50 | **(40·7%)** | 486 | **(27·2%)** | 0·0010 |
| Rheumatology | 44 | **(7·69%)** | 195 | **(12·7%)** | 57 | | **(18·3%)** | 296 | **(12·2%)** | 0·0000 | 47 | **(7·63%)** | 142 | **(13·6%)** | 30 | **(24·4%)** | 219 | **(12·3%)** | 0·0000 |
| Type II Diabetes | 83 | **(14·5%)** | 307 | **(20·0%)** | 98 | | **(31·4%)** | 488 | **(20·2%)** | 0·0000 | 88 | **(14·3%)** | 234 | **(22·4%)** | 39 | **(31·7%)** | 361 | **(20·2%)** | 0·0000 |
| **Number of Co-morbidities** |  |  |  |  |  | |  |  |  |  |  |  |  |  |  |  |  |  |  |
| None | 191 | **(33·4%)** | 361 | **(23·5%)** | 44 | | **(14·1%)** | 596 | **(24·6%)** | 0·0000 | 195 | **(31·7%)** | 211 | **(20·2%)** | 14 | **(11·4%)** | 420 | **(23·5%)** | 0·0000 |
| 1 comorbidity | 135 | **(23·6%)** | 345 | **(22·5%)** | 46 | | **(14·7%)** | 526 | **(21·7%)** |  | 154 | **(25·0%)** | 215 | **(20·6%)** | 17 | **(13·8%)** | 386 | **(21·6%)** |  |
| 2+ comorbidities | 246 | **(43·0%)** | 829 | **(54·0%)** | 222 | | **(71·2%)** | 1297 | **(53·6%)** |  | 267 | **(43·3%)** | 620 | **(59·3%)** | 92 | **(74·8%)** | 979 | **(54·8%)** |  |
| Working full or part time before COVID-19 Illness | 319 | **(71·2%)** | 686 | **(53·9%)** | 88 | | **(31·5%)** | 1093 | **(54·7%)** | 0·0000 | 362 | **(70·2%)** | 421 | **(47·8%)** | 36 | **(32·7%)** | 819 | **(54·3%)** | 0·0000 |
| **BMI Category** |  |  |  |  |  | |  |  |  | 0·4870 |  |  |  |  |  |  |  |  | 0·2380 |
| Underweight (<18·5) | <5 | **(<2%)** | 5 | **(0·33%)** | <5 | | **(<2%)** | 8 | **(0·33%)** |  | 0 | **(0·00%)** | 6 | **(0·57%)** | 0 | **(0·00%)** | 6 | **(0·34%)** |  |
| Normal weight (18·5 to 24·9) | 60 | **(10·5%)** | 145 | **(9·45%)** | 34 | | **(10·9%)** | 239 | **(9·88%)** |  | 66 | **(10·7%)** | 101 | **(9·66%)** | 14 | **(11·4%)** | 181 | **(10·1%)** |  |
| Overweight (25 to 29·9) | 170 | **(29·7%)** | 441 | **(28·7%)** | 82 | | **(26·3%)** | 693 | **(28·6%)** |  | 189 | **(30·7%)** | 306 | **(29·3%)** | 29 | **(23·6%)** | 524 | **(29·4%)** |  |
| Obese (30 to 39·9) | 220 | **(38·5%)** | 642 | **(41·8%)** | 137 | | **(43·9%)** | 999 | **(41·3%)** |  | 243 | **(39·4%)** | 465 | **(44·5%)** | 58 | **(47·2%)** | 766 | **(42·9%)** |  |
| Severe obesity (40+) | 74 | **(12·9%)** | 185 | **(12·1%)** | 54 | | **(17·3%)** | 313 | **(12·9%)** |  | 77 | **(12·5%)** | 136 | **(13·0%)** | 21 | **(17·1%)** | 234 | **(13·1%)** |  |
| **WHO Clinical Progression Scale** |  |  |  |  |  | |  |  |  |  |  |  |  |  |  |  |  |  |  |
| WHO class 4 | 131 | **(22·9%)** | 227 | **(14·8%)** | 34 | | **(10·9%)** | 392 | **(16·2%)** | 0·0000 | 93 | **(15·1%)** | 159 | **(15·2%)** | 17 | **(13·8%)** | 269 | **(15·1%)** | 0·0010 |
| WHO class 5 | 244 | **(42·7%)** | 665 | **(43·3%)** | 124 | | **(39·7%)** | 1033 | **(42·7%)** |  | 282 | **(45·8%)** | 427 | **(40·8%)** | 58 | **(47·2%)** | 767 | **(43·0%)** |  |
| WHO class 6 | 131 | **(22·9%)** | 371 | **(24·2%)** | 63 | | **(20·2%)** | 565 | **(23·4%)** |  | 152 | **(24·7%)** | 259 | **(24·8%)** | 14 | **(11·4%)** | 425 | **(23·8%)** |  |
| WHO class 7 -9 | 66 | **(11·5%)** | 272 | **(17·7%)** | 91 | | **(29·2%)** | 429 | **(17·7%)** |  | 89 | **(14·4%)** | 201 | **(19·2%)** | 34 | **(27·6%)** | 324 | **(18·2%)** |  |

Number (%) with positive response except *Mean [SD] and †Median [IQR]. Percentages are calculated by category after exclusion of missing data for that variable. P values across patient perceived recovery were calculated using a chi-squared test when testing for differences between proportions, ANOVA for normally distributed continuous data and Kruskal Wallis for non-normally distributed continuous data. ^Missing data (age n<5, body mass index n=167, ethnicity n=13, working status before COVID-19 illness n=419)

## Supplementary Table ST3: Univariable ordinal regression for risk of frailty at 5 months and 1 year following hospitalisation

|  |  | 5 Month Visit | | | | 1 Year Visit | | | |
| --- | --- | --- | --- | --- | --- | --- | --- | --- | --- |
|  |  | Odds Ratio | [95% Conf· | Interval] | P>z | Odds Ratio | [95% Conf· | Interval] | P>z |
| Age at admission (years) | <30 | 0·60 | (0·36 - | 1·01) | 0·053 | 0·54 | (0·25 - | 1·15) | 0·108 |
|  | 30-39 | 0·66 | (0·47 - | 0·93) | 0·017 | 0·54 | (0·35 - | 0·81) | 0·003 |
|  | 40-49 | 0·92 | (0·71 - | 1·19) | 0·534 | 0·78 | (0·58 - | 1·05) | 0·106 |
|  | 50-59 | 1·00 |  |  |  | 1·00 |  |  |  |
|  | 60-69 | 1·50 | (1·21 - | 1·86) | <0·001 | 1·31 | (1·03 - | 1·66) | 0·029 |
|  | 70-79 | 2·57 | (1·98 - | 3·35) | <0·001 | 2·40 | (1·78 - | 3·24) | <0·001 |
|  | 80+ | 3·95 | (2·40 - | 6·49) | <0·001 | 4·19 | (2·24 - | 7·84) | <0·001 |
| Sex | Male Sex | 1·00 |  |  |  | 1·00 |  |  |  |
|  | Female Sex | 1·07 | (0·90 - | 1·26) | 0·433 | 1·33 | (1·10 - | 1·61) | 0·003 |
| Hospital Stay (days) |  | 1·02 | (1·02 - | 1·03) | <0·001 | 1·01 | (1·00 - | 1·01) | <0·001 |
| Body Mass Index (kg/m2) |  | 1·01 | (1·00 - | 1·02) | 0·160 | 1·01 | (0·99 - | 1·02) | 0·352 |
| WHO Clinical Progression Scale | WHO severity class 4 | 0·63 | (0·50 - | 0·80) | <0·001 | 1·05 | (0·80 - | 1·39) | 0·725 |
|  | WHO severity class 5 | 1·00 |  |  |  | 1·00 |  |  |  |
|  | WHO severity class 6 | 0·99 | (0·80 - | 1·22) | 0·917 | 0·93 | (0·74 - | 1·18) | 0·565 |
|  | WHO severity class 7 -9 | 1·85 | (1·46 - | 2·33) | <0·001 | 1·54 | (1·18 - | 2·01) | <0·001 |
| Number of Co-morbidities | None | 1·00 |  |  |  | 1·00 |  |  |  |
|  | 1 co-morbidity | 1·31 | (1·04 - | 1·65) | 0·025 | 1·30 | (0·99 - | 1·71) | 0·057 |
|  | 2+ co-morbidities | 2·19 | (1·80 - | 2·67) | <0·001 | 2·39 | (1·90 - | 3·01) | <0·001 |
| Index of Multiple Deprivation Quintile | 5 (Lowest Deprivation) | 1·00 |  |  |  | 1·00 |  |  |  |
|  | 4 | 0·97 | (0·74 - | 1·26) | 0·804 | 0·97 | (0·71 - | 1·32) | 0·831 |
|  | 3 | 1·20 | (0·92 - | 1·57) | 0·177 | 1·36 | (0·93 - | 1·98) | 0·112 |
|  | 2 | 1·38 | (1·08 - | 1·78) | 0·011 | 1·19 | (0·62 - | 2·27) | 0·602 |
|  | 1 (Highest Deprivation) | 1·54 | (1·20 - | 1·99) | 0·001 | 1·80 | (1·08 - | 2·99) | 0·024 |
| Ethnicity | White | 1·00 |  |  |  | 1·00 |  |  |  |
|  | South Asian | 0·84 | (0·65 - | 1·08) | 0·165 | 0·97 | (0·71 - | 1·32) | 0·831 |
|  | Black | 1·30 | (0·95 - | 1·80) | 0·104 | 1·36 | (0·93 - | 1·98) | 0·112 |
|  | Mixed | 0·94 | (0·54 - | 1·63) | 0·821 | 1·19 | (0·62 - | 2·27) | 0·602 |
|  | Other | 1·69 | (1·12 - | 2·56) | 0·013 | 1·80 | (1·08 - | 2·99) | 0·024 |

## Supplementary Table ST4: Multivariable ordinal regression for risk of frailty at 5 months and 1 year following hospitalisation

|  |  | 5 Month Visit | | | | 1 Year Visit | | | |
| --- | --- | --- | --- | --- | --- | --- | --- | --- | --- |
|  |  | Odds Ratio | [95% Conf· | Interval] | P>z | Odds Ratio | [95% Conf· | Interval] | P>z |
| Age at admission (years) | <30 | 0.73 | (0.43 - | 1.25) | 0.252 | 0.56 | (0.26 - | 1.21) | 0.139 |
|  | 30-39 | 0.68 | (0.47 - | 0.97) | 0.033 | 0.59 | (0.39 - | 0.90) | 0.013 |
|  | 40-49 | 0.88 | (0.67 - | 1.14) | 0.324 | 0.85 | (0.64 - | 1.14) | 0.271 |
|  | 50-59 | 1.00 |  |  |  | 1.00 |  |  |  |
|  | 60-69 | 1.42 | (1.13 - | 1.77) | 0.002 | 1.19 | (0.95 - | 1.50) | 0.133 |
|  | 70-79 | 2.77 | (2.11 - | 3.64) | <0.001 | 2.22 | (1.69 - | 2.92) | 0.000 |
|  | 80+ | 4.41 | (2.65 - | 7.36) | <0.001 | 4.04 | (2.37 - | 6.89) | 0.000 |
| Sex | Male Sex | 1.00 |  |  |  | 1.00 |  |  |  |
|  | Female Sex | 1.24 | (1.04 - | 1.48) | 0.015 | 1.49 | (1.24 - | 1.79) | 0.000 |
| WHO Clinical Progression Scale | WHO severity class 4 | 0.81 | (0.63 - | 1.03) | 0.084 | 1.20 | (0.92 - | 1.57) | 0.168 |
|  | WHO severity class 5 | 1.00 |  |  |  | 1.00 |  |  |  |
|  | WHO severity class 6 | 0.97 | (0.78 - | 1.21) | 0.774 | 0.93 | (0.74 - | 1.17) | 0.540 |
|  | WHO severity class 7 -9 | 2.22 | (1.73 - | 2.85) | <0.001 | 1.64 | (1.27 - | 2.13) | 0.000 |
| Number of Co-morbidities | None | 1.00 |  |  |  | 1.00 |  |  |  |
|  | 1 co-morbidity | 1.17 | (0.92 - | 1.49) | 0.205 | 1.14 | (0.88 - | 1.49) | 0.324 |
|  | 2+ co-morbidities | 1.79 | (1.44 - | 2.21) | <0.001 | 1.89 | (1.51 - | 2.36) | 0.000 |
| Index of Multiple Deprivation Quintile | 5 (Lowest Deprivation) | 1.00 |  |  |  | 1.00 |  |  |  |
|  | 4 | 0.97 | (0.74 - | 1.28) | 0.847 | 1.18 | (0.89 - | 1.57) | 0.259 |
|  | 3 | 1.13 | (0.86 - | 1.49) | 0.388 | 1.56 | (1.17 - | 2.09) | 0.002 |
|  | 2 | 1.37 | (1.05 - | 1.79) | 0.019 | 1.42 | (1.07 - | 1.88) | 0.016 |
|  | 1 (Highest Deprivation) | 1.55 | (1.18 - | 2.04) | 0.002 | 1.73 | (1.30 - | 2.31) | 0.000 |
| Ethnicity | White | 1.00 |  |  |  | 1.00 |  |  |  |
|  | South Asian | 1.15 | (0.87 - | 1.52) | 0.315 | 1.22 | (0.90 - | 1.67) | 0.200 |
|  | Black | 0.97 | (0.69 - | 1.36) | 0.860 | 1.04 | (0.72 - | 1.49) | 0.844 |
|  | Mixed | 1.05 | (0.58 - | 1.87) | 0.882 | 1.94 | (1.01 - | 3.71) | 0.047 |
|  | Other | 1.58 | (1.02 - | 2.45) | 0.041 | 1.80 | (1.13 - | 2.88) | 0.014 |
| Time to visit | (days) | 1.00 | (1.00 - | 1.00) | 0.524 | 1.00 | (1.00 - | 1.00) | 0.372 |

Random effects for clustering by site at 5 month visit: variance = 0.105 (95%C.I. 0.0454 – 0.244).

Random effects for clustering by site at 1 year visit: variance = 0.0971 (95%C.I 0.0401 – 0.235).

## Supplementary Figure SF5a: Sensitivity analysis for missing data in the FFP model: Number of participants in each FFP group at 5 months and 1 year including numbers moving between groups during this interval among those with data available for all 5 FFP criteria at both time points.


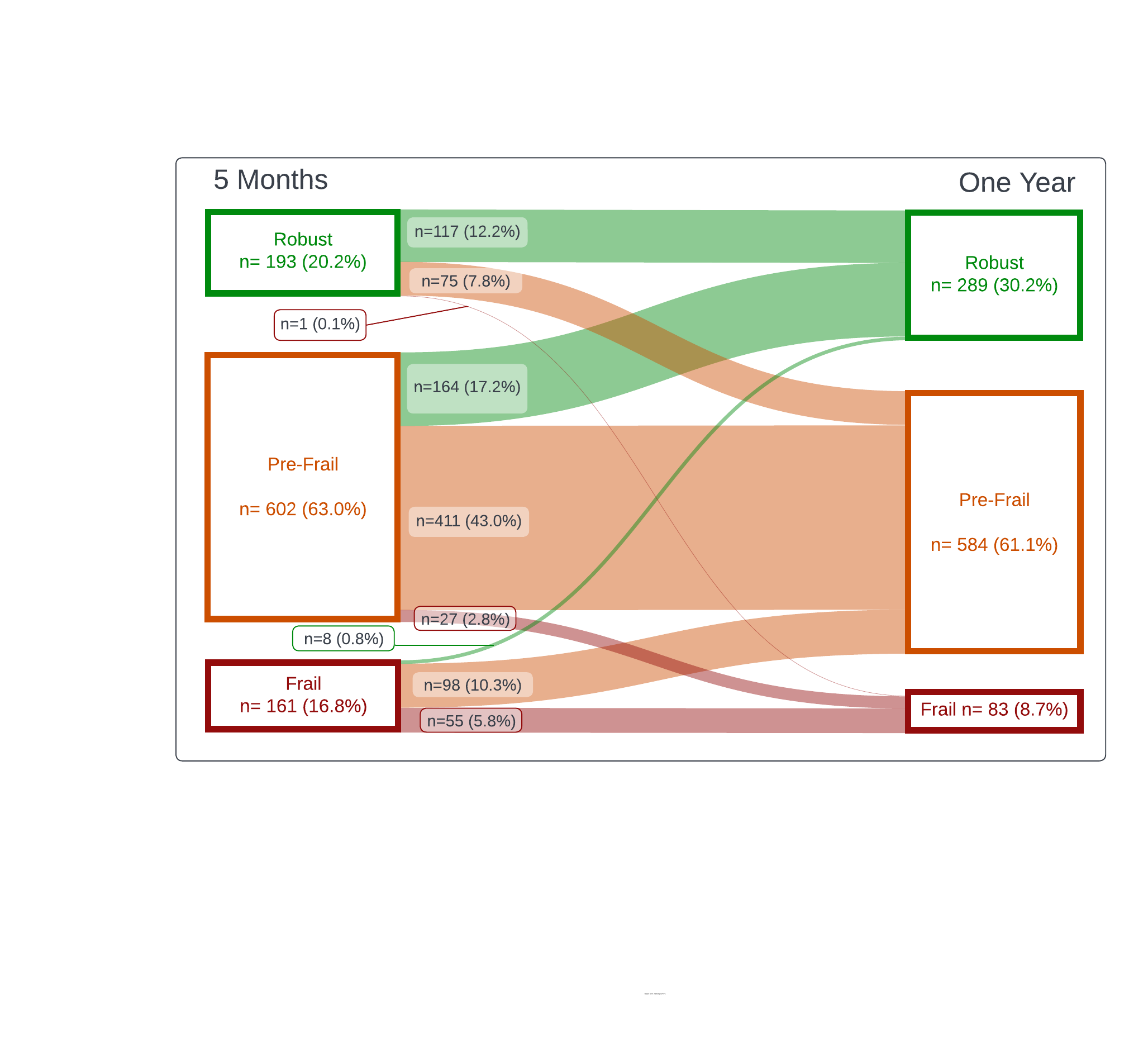


Supplementary Figure SF5b: Sensitivity analysis for missing data in the FFP model: Multivariable ordinal regression for risk of frailty at 5 months and 1 year following hospitalisation among those with data available for all 5 FFP criteria at both time points.
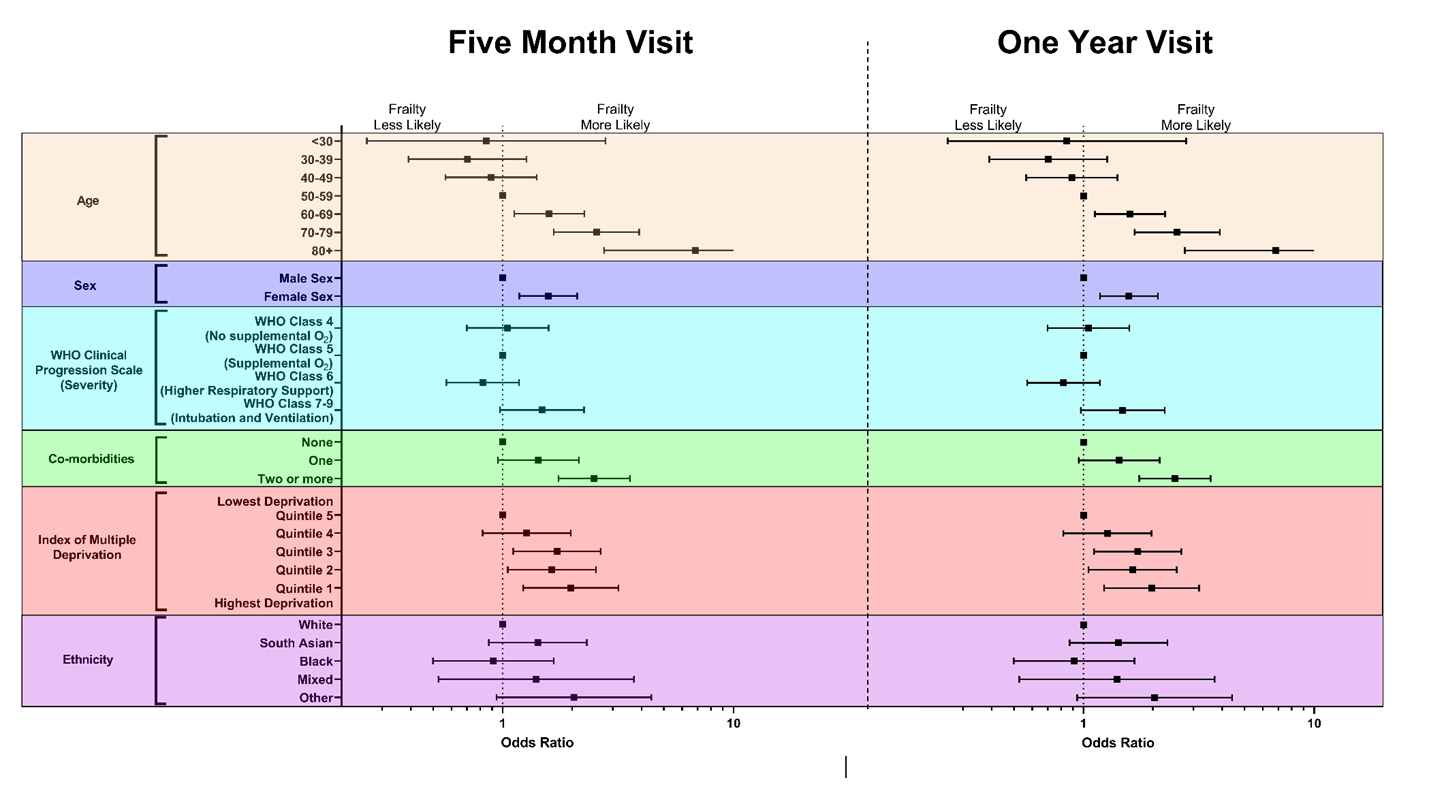


## Supplementary Table ST5c: Sensitivity analysis for missing data in the FFP model: Comparison of baseline characteristics between those with data available for only three of 5 compared to all 5 FFP criteria at both time points.

|  | Participants with  complete data for  three or four  FFP sub-domains | | Participants with  complete data for  all 5  FFP sub-domains | | All included  participants | | p value |
| --- | --- | --- | --- | --- | --- | --- | --- |
| **N** | 776 |  | 1643 |  | 2419 |  |  |
| **Age at admission (years)** ***^** | 58.0 | 13.2 | 57.8 | 12.4 | 57.8 | 12.6 | 0.6836 |
| **Body Mass Index (kg/m^2^)** ***^** | 32.4 | 7.34 | 32.8 | 8.21 | 32.7 | 7.99 | 0.2120 |
| **Hospital Stay (days)** **†** | 8 | 4-16 | 8 | 4-15 | 8 | 4-15 | 0.5201 |
| **Sex** |  |  |  |  |  |  | 0.6100 |
| Female | 305 | 39.3% | 628 | 38.2% | 993 | 41.1% |  |
| Male | 471 | 60.7% | 1,015 | 61.8% | 1,486 | 61.4% |  |
| **Ethnicity^** |  |  |  |  |  |  | 0.9440 |
| White | 573 | 74.5% | 1,222 | 74.6% | 1,795 | 74.6% |  |
| South Asian | 88 | 11.4% | 195 | 11.9% | 283 | 11.8% |  |
| Black | 60 | 7.80% | 113 | 6.90% | 173 | 7.19% |  |
| Mixed | 16 | 2.08% | 36 | 2.20% | 52 | 2.16% |  |
| Other | 32 | 4.16% | 71 | 4.34% | 103 | 4.28% |  |
| **Number of Co-morbidities** |  |  |  |  |  |  | 0.4200 |
| None | 199 | 25.6% | 397 | 24.2% | 596 | 24.6% |  |
| 1 comorbidity | 176 | 22.7% | 350 | 21.3% | 526 | 21.7% |  |
| 2+ comorbidities | 401 | 51.7% | 896 | 54.5% | 1,297 | 53.6% |  |
| Working full or part time before COVID-19 Illness^ | 315 | 50.8% | 778 | 56.4% | 1093 | 54.7% | 0.0210 |
| **WHO Clinical Progression Scale** |  |  |  |  |  |  | 0.5820 |
| WHO class 4 | 127 | 16.4% | 265 | 16.1% | 392 | 16.2% |  |
| WHO class 5 | 345 | 44.5% | 688 | 41.9% | 1,033 | 42.7% |  |
| WHO class 6 | 175 | 22.6% | 390 | 23.7% | 565 | 23.4% |  |
| WHO class 7 -9 | 129 | 16.6% | 300 | 18.3% | 429 | 17.7% |  |

Number (%) with positive response except *Mean [SD] and †Median [IQR]. Percentages are calculated by category after exclusion of missing data for that variable. P values across patient perceived recovery were calculated using a chi-squared test when testing for differences between proportions, ANOVA for normally distributed continuous data and Kruskal Wallis for non-normally distributed continuous data. ^Missing data (age n<5, body mass index n=167, ethnicity n=13, working status before COVID-19 illness n=419)

## Supplementary Figure SF6a: Sensitivity analysis for participants age ≥65 years only: Number of participants in each FFP group at 5 months and 1 year including numbers moving between groups during this interval among participants age ≥65 years only.


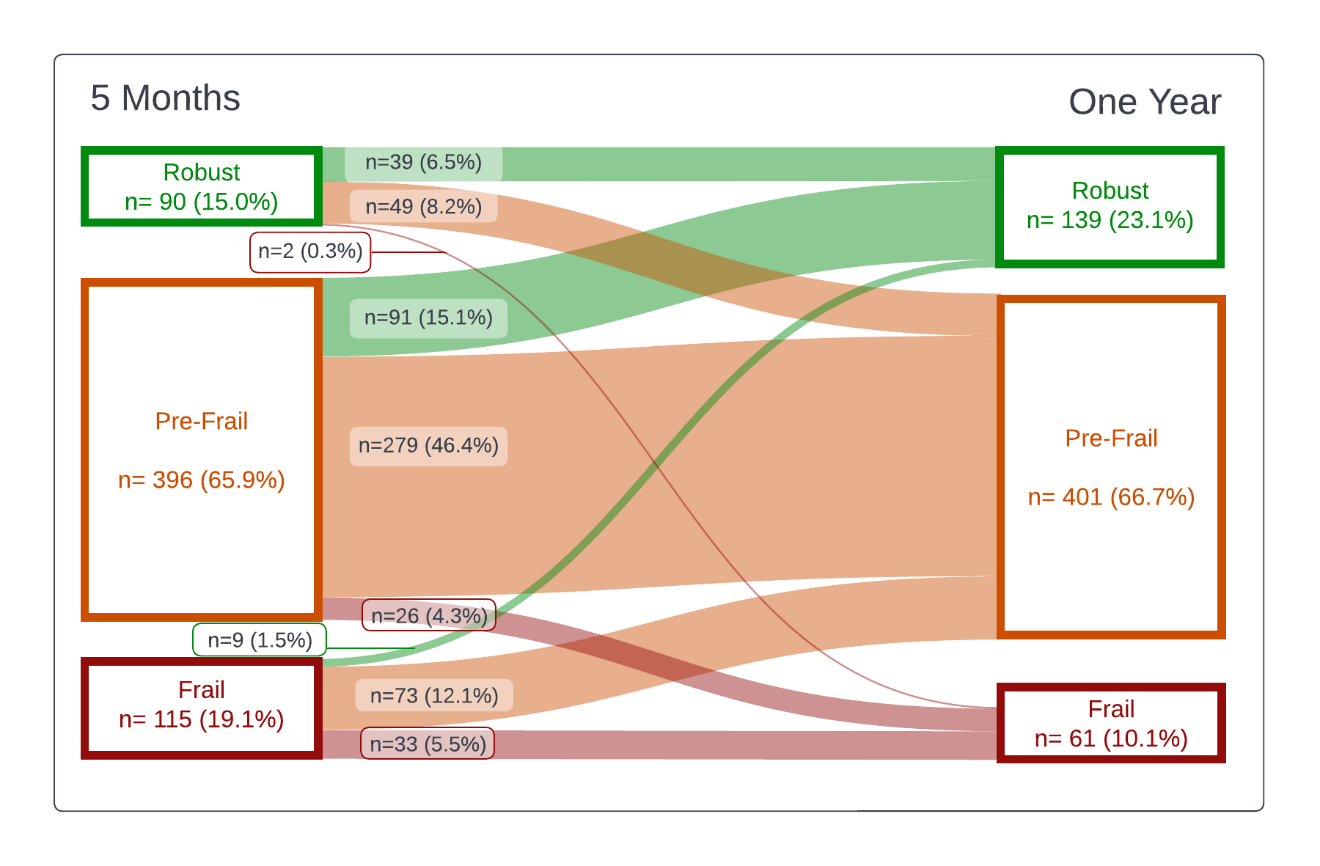


## Supplementary Figure SF6b: Sensitivity analysis for participants age ≥65 years only: Multivariable ordinal regression for risk of frailty at 5 months and 1 year following hospitalisation among participants age ≥65 years only.


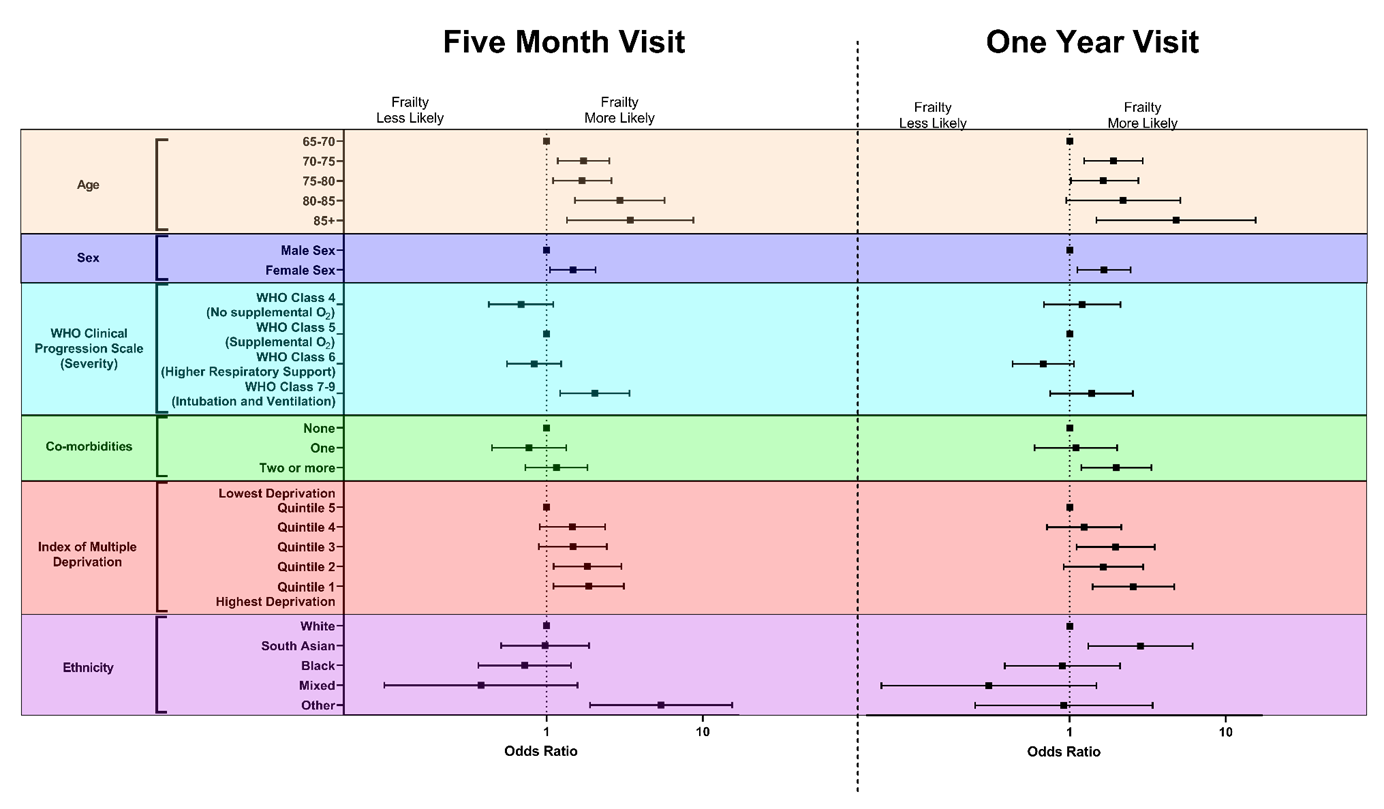


## Supplementary Figure SF7a-d: Sensitivity analysis of transition of participants between frailty status by WHO Severity during acute illness.


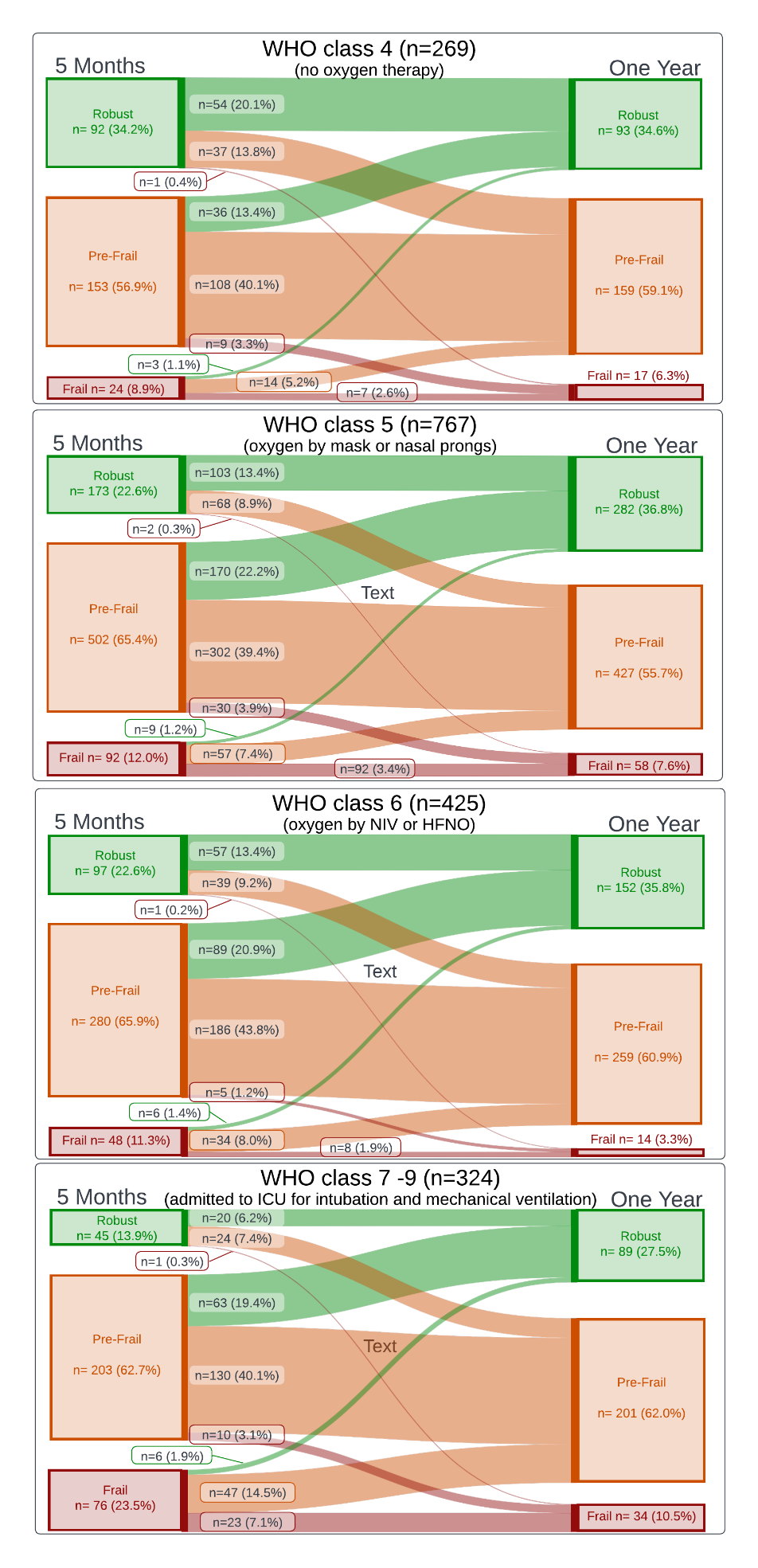


# **References**:

1. Fried LP, Tangen CM, Walston J, et al. Frailty in older adults: evidence for a phenotype. *J Gerontol A Biol Sci Med Sci* 2001; **56**(3): M146-56.

2. Petermann-Rocha F, Hanlon P, Gray SR, et al. Comparison of two different frailty measurements and risk of hospitalisation or death from COVID-19: findings from UK Biobank. *BMC Med* 2020; **18**(1): 355.

3. The General Practice Physical Activity Questionnaire (GPPAQ). 2006. <https://www.nice.org.uk/guidance/cg61/evidence/appendix-j-gppaq-pdf-196701669> (accessed 01/02/2022.

4. English indices of deprivation 2019. <https://www.gov.uk/government/statistics/english-indices-of-deprivation-2019> (accessed 01 May 2022.

5. Office for National Statistics. National Statistics Postcode Lookup (February 2020). February 02, 2020. <https://geoportal.statistics.gov.uk/datasets/national-statistics-postcode-lookup-february-2020> (accessed March 01, 2021.

6. WHO Working Group on the Clinical Characterisation and Management of COVID-19 infection. A minimal common outcome measure set for COVID-19 clinical research. *Lancet Infect Dis* 2020; **20**(8): e192-e7.

7. Devlin NJ, Shah KK, Feng Y, Mulhern B, van Hout B. Valuing health-related quality of life: An EQ-5D-5L value set for England. *Health Econ* 2018; **27**(1): 7-22.
