# Supplementary material for: Prevalence of physical frailty, including risk factors, up to 1 year after hospitalisation for COVID-19 in the UK: a multicentre, longitudinal cohort study
Source: eClinicalMedicine. 2023 Mar 11;57:101896. doi: 10.1016/j.eclinm.2023.101896 (PMC10005893; doi:10.1016/j.eclinm.2023.101896)
Supplement: PHOSP_Collaborative_Group_Authors [file mmc2.docx]

| **First names** | **Surnames** |
| --- | --- |
| K | Abel |
| H | Adamali |
| D | Adeloye |
| O | Adeyemi |
| R | Adrego |
| L A | Aguilar Jimenez |
| S | Ahmad |
| N | Ahmad Haider |
| R | Ahmed |
| N | Ahwireng |
| M | Ainsworth |
| B | Al-Sheklly |
| A | Alamoudi |
| M | Ali |
| M | Aljaroof |
| AM | All |
| L | Allan |
| R J | Allen |
| L | Allerton |
| L | Allsop |
| P | Almeida |
| D | Altmann |
| M | Alvarez Corral |
| S | Amoils |
| D | Anderson |
| C | Antoniades |
| G | Arbane |
| A | Arias |
| C | Armour |
| L | Armstrong |
| N | Armstrong |
| D | Arnold |
| H | Arnold |
| A | Ashish |
| A | Ashworth |
| M | Ashworth |
| S | Aslani |
| H | Assefa-Kebede |
| C | Atkin |
| P | Atkin |
| R | Aul |
| H | Aung |
| L | Austin |
| C | Avram |
| A | Ayoub |
| M | Babores |
| R | Baggott |
| J | Bagshaw |
| D | Baguley |
| L | Bailey |
| J K | Baillie |
| S | Bain |
| M | Bakali |
| M | Bakau |
| E | Baldry |
| D | Baldwin |
| M | Baldwin |
| C | Ballard |
| A | Banerjee |
| B | Bang |
| R E | Barker |
| L | Barman |
| S | Barratt |
| F | Barrett |
| D | Basire |
| N | Basu |
| M | Bates |
| A | Bates |
| R | Batterham |
| H | Baxendale |
| H | Bayes |
| M | Beadsworth |
| P | Beckett |
| M | Beggs |
| M | Begum |
| P | Beirne |
| D | Bell |
| R | Bell |
| K | Bennett |
| E | Beranova |
| A | Bermperi |
| A | Berridge |
| C | Berry |
| S | Betts |
| E | Bevan |
| K | Bhui |
| M | Bingham |
| K | Birchall |
| L | Bishop |
| K | Bisnauthsing |
| J | Blaikely |
| A | Bloss |
| A | Bolger |
| C E | Bolton |
| J | Bonnington |
| A | Botkai |
| C | Bourne |
| M | Bourne |
| K | Bramham |
| L | Brear |
| G | Breen |
| J | Breeze |
| A | Briggs |
| E | Bright |
| C E | Brightling |
| S | Brill |
| K | Brindle |
| L | Broad |
| A | Broadley |
| C | Brookes |
| M | Broome |
| A | Brown |
| A | Brown |
| J | Brown |
| J | Brown |
| J S | Brown |
| M | Brown |
| M | Brown |
| V | Brown |
| T | Brugha |
| N | Brunskill |
| M | Buch |
| P | Buckley |
| A | Bularga |
| E | Bullmore |
| L | Burden |
| T | Burdett |
| D | Burn |
| G | Burns |
| A | Burns |
| J | Busby |
| R | Butcher |
| A | Butt |
| S | Byrne |
| P | Cairns |
| P C | Calder |
| E | Calvelo |
| H | Carborn |
| B | Card |
| C | Carr |
| L | Carr |
| G | Carson |
| P | Carter |
| A | Casey |
| M | Cassar |
| J | Cavanagh |
| M | Chablani |
| T | Chalder |
| J D | Chalmers |
| R C | Chambers |
| F | Chan |
| K M | Channon |
| K | Chapman |
| A | Charalambou |
| N | Chaudhuri |
| A | Checkley |
| J | Chen |
| Y | Cheng |
| L | Chetham |
| C | Childs |
| E R | Chilvers |
| H | Chinoy |
| A | Chiribiri |
| K | Chong-James |
| G | Choudhury |
| N | Choudhury |
| P | Chowienczyk |
| C | Christie |
| M | Chrystal |
| D | Clark |
| C | Clark |
| J | Clarke |
| S | Clohisey |
| G | Coakley |
| Z | Coburn |
| S | Coetzee |
| J | Cole |
| C | Coleman |
| F | Conneh |
| D | Connell |
| B | Connolly |
| L | Connor |
| A | Cook |
| B | Cooper |
| J | Cooper |
| S | Cooper |
| D | Copeland |
| T | Cosier |
| M | Coulding |
| C | Coupland |
| E | Cox |
| T | Craig |
| P | Crisp |
| D | Cristiano |
| M G | Crooks |
| A | Cross |
| I | Cruz |
| P | Cullinan |
| D | Cuthbertson |
| L | Daines |
| M | Dalton |
| P | Daly |
| A | Daniels |
| P | Dark |
| J | Dasgin |
| A | David |
| C | David |
| E | Davies |
| F | Davies |
| G | Davies |
| G A | Davies |
| K | Davies |
| M J | Davies |
| J | Dawson |
| E | Daynes |
| A | De Soyza |
| B | Deakin |
| A | Deans |
| C | Deas |
| J | Deery |
| S | Defres |
| A | Dell |
| K | Dempsey |
| E | Denneny |
| J | Dennis |
| A | Dewar |
| R | Dharmagunawardena |
| N | Diar-Bakerly |
| C | Dickens |
| A | Dipper |
| S | Diver |
| S N | Diwanji |
| M | Dixon |
| R | Djukanovic |
| H | Dobson |
| S L | Dobson |
| A B | Docherty |
| A | Donaldson |
| T | Dong |
| N | Dormand |
| A | Dougherty |
| R | Dowling |
| S | Drain |
| K | Draxlbauer |
| K | Drury |
| H J C | drury |
| P | Dulawan |
| A | Dunleavy |
| S | Dunn |
| C | Dupont |
| J | Earley |
| N | Easom |
| C | Echevarria |
| S | Edwards |
| C | Edwardson |
| H | El-Taweel |
| A | Elliott |
| K | Elliott |
| Y | Ellis |
| A | Elmer |
| O | Elneima |
| D | Evans |
| H | Evans |
| J | Evans |
| R | Evans |
| R A | Evans |
| R I | Evans |
| T | Evans |
| C | Evenden |
| L | Evison |
| L | Fabbri |
| S | Fairbairn |
| A | Fairman |
| K | Fallon |
| D | Faluyi |
| C | Favager |
| T | Fayzan |
| J | Featherstone |
| T | Felton |
| J | Finch |
| S | Finney |
| J | Finnigan |
| L | Finnigan |
| H | Fisher |
| S | Fletcher |
| R | Flockton |
| M | Flynn |
| H | Foot |
| D | Foote |
| A | Ford |
| D | Forton |
| E | Fraile |
| C | Francis |
| R | Francis |
| S | Francis |
| A | Frankel |
| E | Fraser |
| R | Free |
| N | French |
| X | Fu |
| J | Fuld |
| J | Furniss |
| L | Garner |
| N | Gautam |
| J R | Geddes |
| J | George |
| P | George |
| M | Gibbons |
| M | Gill |
| L | Gilmour |
| F | Gleeson |
| J | Glossop |
| S | Glover |
| N | Goodman |
| C | Goodwin |
| B | Gooptu |
| H | Gordon |
| T | Gorsuch |
| M | Greatorex |
| P L | Greenhaff |
| W | Greenhalf |
| A | Greenhalgh |
| N J | Greening |
| J | Greenwood |
| H | Gregory |
| R | Gregory |
| D | Grieve |
| D | Griffin |
| L | Griffiths |
| A-M | Guerdette |
| B | Guillen Guio |
| M | Gummadi |
| A | Gupta |
| S | Gurram |
| E | Guthrie |
| [Z](mailto:Zoe.Guy@york.nhs.uk) | Guy |
| H | H Henson |
| K | Hadley |
| A | Haggar |
| K | Hainey |
| B | Hairsine |
| P | Haldar |
| I | Hall |
| L | Hall |
| M | Halling-Brown |
| R | Hamil |
| A | Hancock |
| K | Hancock |
| N A | Hanley |
| S | Haq |
| H E | Hardwick |
| E | Hardy |
| T | Hardy |
| B | Hargadon |
| K | Harrington |
| E | Harris |
| V C | Harris |
| E M | Harrison |
| P | Harrison |
| N | Hart |
| A | Harvey |
| M | Harvey |
| M | Harvie |
| L | Haslam |
| M | Havinden-Williams |
| J | Hawkes |
| N | Hawkings |
| J | Haworth |
| A | Hayday |
| M | Haynes |
| J | Hazeldine |
| T | Hazelton |
| L G | Heaney |
| C | Heeley |
| J L | Heeney |
| M | Heightman |
| S | Heller |
| M | Henderson |
| L | Hesselden |
| M | Hewitt |
| V | Highett |
| T | Hillman |
| T | Hiwot |
| L P | Ho |
| A | Hoare |
| M | Hoare |
| J | Hockridge |
| P | Hogarth |
| A | Holbourn |
| S | Holden |
| L | Holdsworth |
| D | Holgate |
| M | Holland |
| L | Holloway |
| K | Holmes |
| M | Holmes |
| B | Holroyd-Hind |
| L | Holt |
| A | Hormis |
| A | Horsley |
| A | Hosseini |
| M | Hotopf |
| L | Houchen-Wolloff |
| K | Howard |
| L S | Howard |
| A | Howell |
| E | Hufton |
| A D | Hughes |
| J | Hughes |
| R | Hughes |
| A | Humphries |
| N | Huneke |
| E | Hurditch |
| J | Hurst |
| M | Husain |
| T | Hussell |
| J | Hutchinson |
| W | Ibrahim |
| F | Ilyas |
| J | Ingham |
| L | Ingram |
| D | Ionita |
| K | Isaacs |
| K | Ismail |
| T | Jackson |
| J | Jacob |
| W Y | James |
| W | Jang |
| C | Jarman |
| I | Jarrold |
| H | Jarvis |
| R | Jastrub |
| B | Jayaraman |
| R G | Jenkins |
| P | Jezzard |
| K | Jiwa |
| C | Johnson |
| S | Johnson |
| D | Johnston |
| C J | Jolley |
| D | Jones |
| G | Jones |
| H | Jones |
| H | Jones |
| I | Jones |
| L | Jones |
| M G | Jones |
| S | Jones |
| S | Jose |
| T | Kabir |
| G | Kaltsakas |
| V | Kamwa |
| N | Kanellakis |
| s | Kaprowska |
| Z | Kausar |
| N | Keenan |
| S | Kelly |
| G | Kemp |
| S | Kerr |
| H | Kerslake |
| A L | Key |
| F | Khan |
| K | Khunti |
| S | Kilroy |
| B | King |
| C | King |
| L | Kingham |
| J | Kirk |
| P | Kitterick |
| P | Klenerman |
| L | Knibbs |
| S | Knight |
| A | Knighton |
| O | Kon |
| S | Kon |
| S S | Kon |
| S | Koprowska |
| A | Korszun |
| I | Koychev |
| C | Kurasz |
| P | Kurupati |
| C | Laing |
| H | Lamlum |
| G | Landers |
| C | Langenberg |
| D | Lasserson |
| L | Lavelle-Langham |
| A | Lawrie |
| C | Lawson |
| C | Lawson |
| A | Layton |
| A | Lea |
| O C | Leavy |
| D | Lee |
| J-H | Lee |
| E | Lee |
| K | Leitch |
| R | Lenagh |
| D | Lewis |
| J | Lewis |
| K E | Lewis |
| V | Lewis |
| N | Lewis-Burke |
| X | Li |
| T | Light |
| L | Lightstone |
| W | Lilaonitkul |
| L | Lim |
| S | Linford |
| A | Lingford-Hughes |
| M | Lipman |
| K | Liyanage |
| A | Lloyd |
| S | Logan |
| D | Lomas |
| N I | Lone |
| R | Loosley |
| J M | Lord |
| H | Lota |
| W | Lovegrove |
| A | Lucey |
| E | Lukaschuk |
| A | Lye |
| C | Lynch |
| S | MacDonald |
| G | MacGowan |
| I | Macharia |
| J | Mackie |
| L | Macliver |
| S | Madathil |
| G | Madzamba |
| N | Magee |
| M M | Magtoto |
| N | Mairs |
| N | Majeed |
| E | Major |
| F | Malein |
| M | Malim |
| G | Mallison |
| W D-C | Man |
| S | Mandal |
| K | Mangion |
| C | Manisty |
| R | Manley |
| K | March |
| S | Marciniak |
| P | Marino |
| M | Mariveles |
| M | Marks |
| E | Marouzet |
| S | Marsh |
| B | Marshall |
| M | Marshall |
| J | Martin |
| A | Martineau |
| L M | Martinez |
| N | Maskell |
| D | Matila |
| W | Matimba-Mupaya |
| L | Matthews |
| A | Mbuyisa |
| S | McAdoo |
| H | McAllister-Williams |
| A | McArdle |
| P | McArdle |
| D | McAulay |
| G P | McCann |
| J | McCormick |
| W | McCormick |
| P | McCourt |
| L | McGarvey |
| C | McGee |
| K | Mcgee |
| J | McGinness |
| K | McGlynn |
| A | McGovern |
| H | McGuinness |
| I B | McInnes |
| J | McIntosh |
| E | McIvor |
| K | McIvor |
| L | McLeavey |
| A | McMahon |
| M J | McMahon |
| L | McMorrow |
| T | Mcnally |
| M | McNarry |
| J | McNeill |
| A | McQueen |
| H | McShane |
| C | Mears |
| C | Megson |
| S | Megson |
| P | Mehta |
| J | Meiring |
| L | Melling |
| M | Mencias |
| D | Menzies |
| M | Merida Morillas |
| A | Michael |
| C | Miller |
| L | Milligan |
| C | Mills |
| G | Mills |
| N L | Mills |
| L | Milner |
| S | Misra |
| J | Mitchell |
| A | Mohamed |
| N | Mohamed |
| S | Mohammed |
| P L | Molyneaux |
| W | Monteiro |
| S | Moriera |
| A | Morley |
| L | Morrison |
| R | Morriss |
| A | Morrow |
| A J | Moss |
| P | Moss |
| K | Motohashi |
| N | Msimanga |
| E | Mukaetova-Ladinska |
| U | Munawar |
| J | Murira |
| U | Nanda |
| H | Nassa |
| M | Nasseri |
| A | Neal |
| R | Needham |
| P | Neill |
| S | Neubauer |
| D E | Newby |
| H | Newell |
| T | Newman |
| J | Newman |
| A | Newton-Cox |
| T | Nicholson |
| D | Nicoll |
| A | Nikolaidis |
| C M | Nolan |
| M J | Noonan |
| C | Norman |
| P | Novotny |
| J | Nunag |
| L | Nwafor |
| U | Nwanguma |
| J | Nyaboko |
| C | O'Brien |
| K | O'Donnell |
| D | O'Regan |
| L | O’Brien |
| N | Odell |
| G | Ogg |
| O | Olaosebikan |
| C | Oliver |
| Z | Omar |
| P J M | Openshaw |
| L | Orriss-Dib |
| L | Osborne |
| R | Osbourne |
| M | Ostermann |
| C | Overton |
| J | Owen |
| J | Oxton |
| J | Pack |
| E | Pacpaco |
| S | Paddick |
| S | Painter |
| A | Pakzad |
| S | Palmer |
| P | Papineni |
| K | Paques |
| K | Paradowski |
| M | Pareek |
| D | Parekh |
| H | Parfrey |
| C | Pariante |
| S | Parker |
| M | Parkes |
| J | Parmar |
| S | Patale |
| B | Patel |
| M | Patel |
| S | Patel |
| D | Pattenadk |
| M | Pavlides |
| S | Payne |
| L | Pearce |
| J E | Pearl |
| D | Peckham |
| J | Pendlebury |
| Y | Peng |
| C | Pennington |
| I | Peralta |
| E | Perkins |
| Z | Peterkin |
| T | Peto |
| N | Petousi |
| J | Petrie |
| P | Pfeffer |
| J | Phipps |
| J | Pimm |
| K | Piper Hanley |
| R | Pius |
| H | Plant |
| S | Plein |
| T | Plekhanova |
| M | Plowright |
| K | Poinasamy |
| O | Polgar |
| L | Poll |
| J C | Porter |
| J | Porter |
| S | Portukhay |
| N | Powell |
| A | Prabhu |
| J | Pratt |
| A | Price |
| C | Price |
| C | Price |
| D | Price |
| L | Price |
| L | Price |
| A | Prickett |
| J | Propescu |
| S | Prosper |
| S | Pugmire |
| S | Quaid |
| J | Quigley |
| J | Quint |
| H | Qureshi |
| I N | Qureshi |
| K | Radhakrishnan |
| N M | Rahman |
| M | Ralser |
| B | Raman |
| A | Ramos |
| H | Ramos |
| J | Rangeley |
| B | Rangelov |
| L | Ratcliffe |
| P | Ravencroft |
| A | Reddington |
| R | Reddy |
| A | Reddy |
| H | Redfearn |
| D | Redwood |
| A | Reed |
| M | Rees |
| T | Rees |
| K | Regan |
| W | Reynolds |
| C | Ribeiro |
| A | Richards |
| E | Richardson |
| M | Richardson |
| P | Rivera-Ortega |
| K | Roberts |
| E | Robertson |
| E | Robinson |
| L | Robinson |
| L | Roche |
| C | Roddis |
| J | Rodger |
| A | Ross |
| G | Ross |
| J | Rossdale |
| A | Rostron |
| A | Rowe |
| A | Rowland |
| J | Rowland |
| M J | Rowland |
| S L | Rowland-Jones |
| K | Roy |
| M | Roy |
| I | Rudan |
| R | Russell |
| E | Russell |
| G | Saalmink |
| R | Sabit |
| E K | Sage |
| T | Samakomva |
| N | Samani |
| C | Sampson |
| K | Samuel |
| R | Samuel |
| A | Sanderson |
| E | Sapey |
| D | Saralaya |
| J | Sargant |
| C | Sarginson |
| T | Sass |
| N | Sattar |
| K | Saunders |
| R M | Saunders |
| P | Saunders |
| L C | Saunders |
| H | Savill |
| W | Saxon |
| A | Sayer |
| J | Schronce |
| W | Schwaeble |
| J T | Scott |
| K | Scott |
| N | Selby |
| M G | Semple |
| M | Sereno |
| T A | Sewell |
| A | Shah |
| K | Shah |
| P | Shah |
| M | Shankar-Hari |
| M | Sharma |
| C | Sharpe |
| M | Sharpe |
| S | Shashaa |
| A | Shaw |
| K | Shaw |
| V | Shaw |
| A | Sheikh |
| S | Shelton |
| L | Shenton |
| K | Shevket |
| A | Shikotra |
| J | Short |
| S | Siddique |
| S | Siddiqui |
| J | Sidebottom |
| L | Sigfrid |
| G | Simons |
| J | Simpson |
| N | Simpson |
| A | Singapuri |
| C | Singh |
| S | Singh |
| S J | Singh |
| D | Sissons |
| J | Skeemer |
| K | Slack |
| A | Smith |
| D | Smith |
| S | Smith |
| J | Smith |
| L | Smith |
| M | Soares |
| T S | Solano |
| R | Solly |
| AR | Solstice |
| T | Soulsby |
| D | Southern |
| D | Sowter |
| M | Spears |
| L G | Spencer |
| F | Speranza |
| L | Stadon |
| S | Stanel |
| N | Steele |
| M | Steiner |
| D | Stensel |
| G | Stephens |
| L | Stephenson |
| M | Stern |
| I | Stewart |
| R | Stimpson |
| S | Stockdale |
| J | Stockley |
| W | Stoker |
| R | Stone |
| W | Storrar |
| A | Storrie |
| K | Storton |
| E | Stringer |
| S | Strong-Sheldrake |
| N | Stroud |
| C | Subbe |
| C L | Sudlow |
| Z | Suleiman |
| C | Summers |
| C | Summersgill |
| D | Sutherland |
| D L | Sykes |
| R | Sykes |
| N | Talbot |
| A L | Tan |
| L | Tarusan |
| V | Tavoukjian |
| A | Taylor |
| C | Taylor |
| J | Taylor |
| A | Te |
| H | Tedd |
| CJ | Tee |
| J | Teixeira |
| H | Tench |
| S | Terry |
| S | Thackray-Nocera |
| F | Thaivalappil |
| B | Thamu |
| D | Thickett |
| C | Thomas |
| D C | Thomas |
| S | Thomas |
| A K | Thomas |
| T | Thomas-Woods |
| T | Thompson |
| A A R | Thompson |
| T | Thornton |
| M | Thorpe |
| R S | Thwaites |
| J | Tilley |
| N | Tinker |
| G F | Tiongson |
| M | Tobin |
| J | Tomlinson |
| C | Tong |
| M | Toshner |
| R | Touyz |
| K A | Tripp |
| E | Tunnicliffe |
| A | Turnbull |
| E | Turner |
| S | Turner |
| V | Turner |
| K | Turner |
| S | Turney |
| L | Turtle |
| H | Turton |
| J | Ugoji |
| R | Ugwuoke |
| R | Upthegrove |
| J | Valabhji |
| M | Ventura |
| J | Vere |
| C | Vickers |
| B | Vinson |
| E | Wade |
| P | Wade |
| L V | Wain |
| T | Wainwright |
| L O | Wajero |
| S | Walder |
| S | Walker |
| S | Walker |
| E | Wall |
| T | Wallis |
| S | Walmsley |
| J A | Walsh |
| S | Walsh |
| L | Warburton |
| T J C | Ward |
| K | Warwick |
| H | Wassall |
| S | Waterson |
| E | Watson |
| L | Watson |
| J | Watson |
| J | Weir McCall |
| C | Welch |
| H | Welch |
| B | Welsh |
| S | Wessely |
| S | West |
| H | Weston |
| H | Wheeler |
| S | White |
| V | Whitehead |
| J | Whitney |
| S | Whittaker |
| B | Whittam |
| V | Whitworth |
| A | Wight |
| J | Wild |
| M | Wilkins |
| D | Wilkinson |
| B | Williams |
| N | Williams |
| N | Williams |
| J | Williams |
| S A | Williams-Howard |
| M | Willicombe |
| G | Willis |
| J | Willoughby |
| A | Wilson |
| D | Wilson |
| I | Wilson |
| N | Window |
| M | Witham |
| R | Wolf-Roberts |
| C | Wood |
| F | Woodhead |
| J | Woods |
| D G | Wootton |
| J | Wormleighton |
| J | Worsley |
| D | Wraith |
| C | Wrey Brown |
| C | Wright |
| L | Wright |
| S | Wright |
| J | Wyles |
| I | Wynter |
| M | Xu |
| N | Yasmin |
| S | Yasmin |
| T | Yates |
| K P | Yip |
| B | Young |
| S | Young |
| A | Young |
| A J | Yousuf |
| A | Zawia |
| L | Zeidan |
| B | Zhao |
| B | Zheng |
| O | Zongo |
